# Flash Communication: Pd_2_Zn_2_ Clusters from the Reduction of Palladium(II) Dichloride Precursors with Metallic Zinc

**DOI:** 10.1021/acs.organomet.4c00506

**Published:** 2025-03-08

**Authors:** Georgina Rai, Martí Garçon, Philip W. Miller, Mark R. Crimmin

**Affiliations:** Molecular Sciences Research Hub, Imperial College London, 82 Wood Lane, Shepherds Bush, London W12 0BZ, U.K.

## Abstract

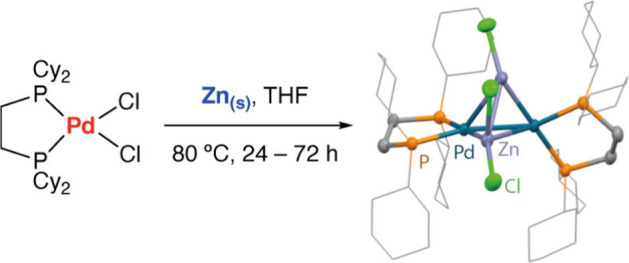

We report the synthesis and solid-state characterization of two unusual Pd_2_Zn_2_ clusters formed from the partial reduction of [PdL_2_Cl_2_] precursors (L_2_ = dcpe or dppe) with metallic zinc. The new clusters have been characterized by single crystal X-ray diffraction and contain a Pd_2_Zn_2_Cl_3_ core capped by two chelating phosphine ligands with Zn in the formal +1.5 oxidation state. While they possess a near tetrahedral arrangement of metal ions, calculations and bonding analysis (NBO, AIM) suggest that there is limited Zn- - -Zn bonding in these species. Characterization in the solution state is suggestive of dynamic behavior on dissolution, with both diamagnetic and paramagnetic species observed by NMR and EPR spectroscopy. One of these Pd_2_Zn_2_ clusters was shown to be an effective precursor for the homocoupling of an aryl bromide.

Palladium clusters, Pd_*n*_ (*n* ≥ 2), are of interest in catalytic processes. Known for over 50 years, palladium clusters have been studied as a means to understand the mechanisms of hydrogenation and cross-coupling reactions.^1−8^ In particular, species that contain both palladium and an electropositive metal (e.g., Mg, Zn, Sn) have received attention due to their potential role as intermediates in transmetalation steps. In the case of Negishi coupling, complexes containing defined Pd–Zn bonds have been isolated;^9,10^ these include a handful of crystallographically characterized clusters containing PdZn_2_,^11^ Pd_2_Zn,^12^ Pd_2_Zn_3_,^13^ Pd_3_Zn_6_,^14^ and Pd_4_Zn_8_^15^ metallic motifs. Our understanding of such heterometallic clusters is still in its infancy. Further developments are necessary to understand both the nature of metal–metal bonding and their reactivity. Herein we report the synthesis of two unusual heterometallic clusters based on a Pd_2_Zn_2_ core. These species have been characterized in the solid state, and their electronic structures have been investigated using computational methods. Preliminary evaluation of their solution behavior suggests that they do not remain intact upon dissolution. While the precise nature of the solution species is yet to be determined, we show that one of these clusters is an effective precursor for the homocoupling an aryl bromide.

Treatment of [Pd(dcpe)Cl_2_] (**1a**, dcpe = 1,2-bis(dicyclohexylphosphino)ethane) with metallic Zn dust at 80 °C in THF furnished cluster **2a** after 72 h. Due to the harsh conditions of the heterogeneous reduction, crystals suitable for X-ray diffraction were produced in very low but reproducible yield, along with Pd(0) black and undetermined byproducts. Treatment of [Pd(dcpp)Cl_2_] (**1b**, dcpp = 1,2-bis(dicyclohexylphosphino)propane) with metallic Zn dust at 80 °C in THF lead to the formation of cluster **2b** after 24 h, in a 6% isolated yield. Albeit a cleaner reaction, Pd(0) black is still produced. Cluster **2b** decomposes over time more readily than **2a**. Once isolated, both clusters are stable if kept as solids at–35 °C under an N_2_ atmosphere (Scheme 1).

**Scheme 1:**
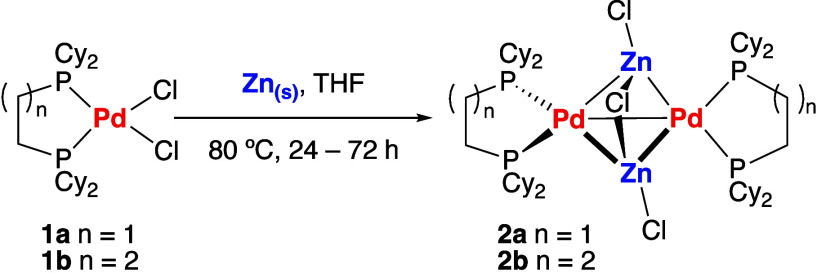
Synthesis of Pd_2_Zn_2_ Clusters **2a** and **2b**

**2a** crystallizes in the *P*2_1_/*n* space group, while **2b** crystallizes in the *P*1̅ space group (Figure 1). Their structures are broadly similar. In each case, the core of the cluster is formed from two Pd and two Zn atoms apparently connected in a distorted tetrahedral arrangement. The Pd–Zn bonds are unsymmetrical and each Pd center is connected to two Zn centers by a shorter bond length ranging from 2.4589(6) to 2.4673(8) Å and longer interaction ranging from 2.7080(9) to 2.7963(7) Å. The data suggest each Zn fragment may be more strongly associated with one Pd center than the other. For comparison, the sums of the single-bond covalent radii are 2.38 Å (Pyykkö)^16^ and 2.53 Å (Pauling).^17^ Pd–Zn bond lengths in which a Z-type ZnX_2_ ligand is coordinated to Pd take values between 2.5786(3) and 2.6399(5) Å.^9,18^ The Zn- - -Zn distances are similar for both **2a** and **2b**, taking values of 2.6818(8) and 2.6357(11) Å, respectively; the Pd–Pd separation in **2a** of 2.7981(4) Å is over 0.1 Å shorter than that of **2b** of 2.9033(6) Å. The most closely related known cluster contains a Pd_2_Zn_4_ core, with a much larger Pd–Pd distance of 3.256(1) Å and Zn- - -Zn separations of over 2.854(1) Å.^13^ Three chloride ions of **2a** and **2b** are associated with the two Zn centers, with Zn–Cl bond lengths ranging from 2.4304(13) to 2.4571(13) Å for the bridging and 2.1914(16) to 2.2173(13) Å for terminal ligands. Some subtle structural differences between **2a** and **2b** are seen due to the smaller bite angle of dcpe (88.2(3)–89.0(0)°) compared to that of dcpp (99.4(1)–99.4(3)°).

**Figure 1:**
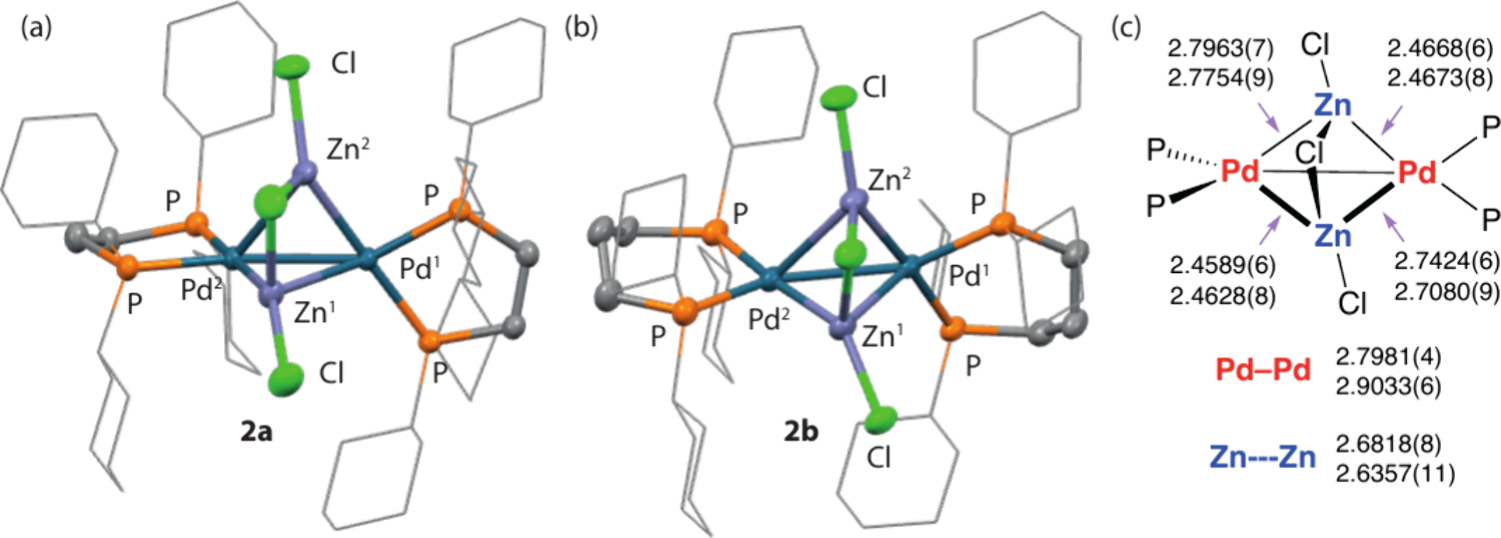
Crystal structures of (a) **2a** and (b) **2b**, along with (c) a line drawing depicting key bond lengths (Å) of **2a** (top value) and **2b** (bottom value).

In the solid state, **2a** and **2b** can be formalized as L_4_Pd_2_Zn_2_X_3_ complexes (L = 2-electron ligand, X = 1-electron ligand) and as such should be odd-electron species. To gain further insight into the structure and bonding of these species, a series of DFT calculations were conducted. Several computational models were explored (Supporting Information), with calculations ultimately being carried out with the M062X functional. Only in the case of definition of a doublet ground state (*S* = 1/2) did the computational model begin to align with the structural data from X-ray diffraction.

Natural bond orbital (NBO) calculations were performed to obtain NPA charges and Wiberg bond indices (WBIs) for **2a** and **2b** (Figure 2a,b).^19,20^ For both clusters, NPA charges are equally highest on the Zn atoms (+0.58 to +0.60). For comparison, the charge on the Zn^2+^ sites in a dimeric zinc(II) dichloride moiety, Zn_2_Cl_4_, is calculated to be +1.47 at the same level of theory. The charge on Pd in **2a** and **2b** is relatively small (−0.25 to −0.28). While care should be taken in correlating calculated charges and formal oxidation states, these values offer useful insight into the bonding and oxidation states of the metals. The charges on Pd are consistent with Pd(0), whereas the charges on Zn are substantially lower than those expected for Zn(II) and could be consistent with a formal oxidation state between Zn(I) and Zn(II). The WBIs suggest that the metal–metal bonding is dominated by electrostatic intermetallic interactions. The WBIs for the Pd–Pd bonds in **2a** and **2b** are 0.24 and 0.20, respectively, reflecting the differing bond lengths of the solid-state structures. Interestingly, the WBIs for Zn–Zn are very close to zero, suggesting very little, if any, interaction. Pd–Zn WBIs are again in good agreement with data obtained from the solid-state structures, whereby two Pd–Zn bonds have WBIs larger than the other (0.26 to 0.28 and 0.09 to 0.13). AIM calculations return bond paths and critical points between the two Pd atoms along with the expected Pd–Zn interactions. For both **2a** and **2b**, no bond critical points were found between the Zn atoms, indicating the limited importance of Zn- - -Zn bonding in these clusters. Values of ρ(*r*) and ∇^2^ρ(*r*) between Pd–Pd and Pd–Zn bonds are all small and positive, consistent with primarily closed-shell, weak bonding interactions (Figure 2c).

**Figure 2:**
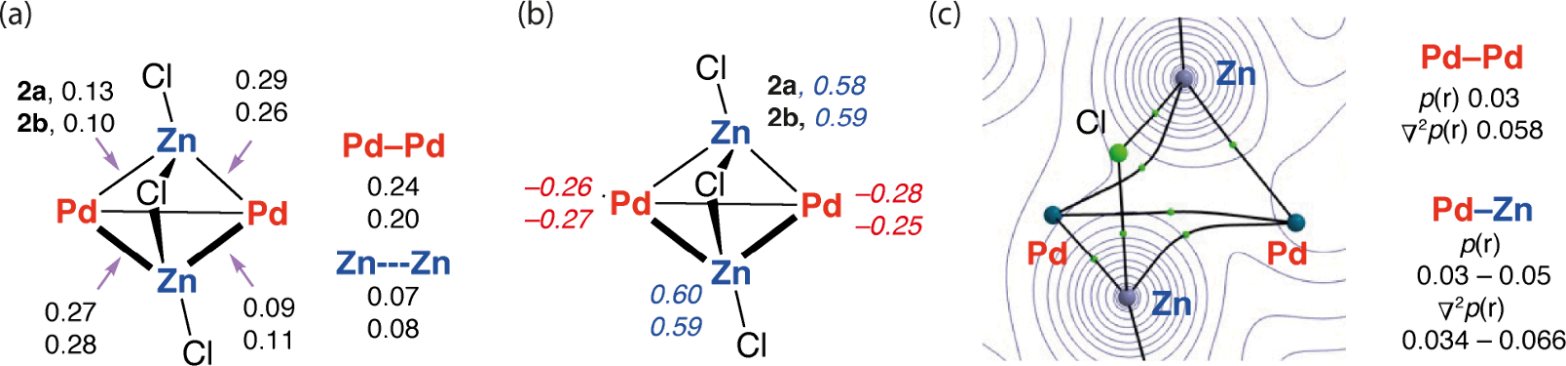
NBO calculations including (a) Wiberg bond indices and (b) NPA charges of **2a** (top value) and **2b** (bottom value), along with (c) an AIM graph of **2b**.

Further support for the compositions of **2a** and **2b** was provided by mass spectrometry data. While the molecular ion or any sensible fragment ions of **2a** could not be identified by ESI mass spectrometry, **2b** showed a signal with a distinctive isotope pattern [M – Cl]^+^ at 1289.2822 *m*/*z*corresponding to the expected mass of [M–Cl]^+^ (1286.2778 *m*/*z*).

The solution behavior of **2a** and **2b** is complex. NMR and EPR spectroscopic data strongly suggest that the cluster does not remain intact in solution but rather fragments into as yet unidentified diamagnetic and paramagnetic species. Upon dissolution a singlet is observed in the ^31^P NMR spectra at δ_^31^P_ = −14.08 and–10.68 ppm for **2a** and **2b**, respectively, along with *J*_C–P_ splitting visible in the ^13^C NMR spectra. For **2b**, continuous wave electron paramagnetic resonance (CW EPR) spectroscopy at 40 K displays a signal with *g* values of *g*_∥_ = 2.01 and *g*_⊥_ = 2.22. While further work is required to unambiguously confirm the dynamic process involved, one possibility is that these clusters fragment into monomeric palladium complexes of the form L_2_PdZnX_2_ and L_2_PdZnX in solution.

Preliminary reactivity studies show that these species have relevance, at least as reservoirs of Pd in cross-coupling reactions. Hence addition of ArBr (Ar = 4-CF_3_C_6_H_4_) to **2b** in C_6_D_6_ solution at 80 °C led to the very slow formation of the homocoupled product Ar–Ar in 60% yield, along with the hydrodebromination product ArH in 34%, after 24 days. The proton in the latter product likely derives from the residual solvent. This homocoupling reaction occurred with the formation of a mixture of palladium(II) side products. Fractional crystallization allowed the identification of [Pd(dcpp)X_4_Zn] (**3**, X = Br, Cl) as components of this mixture. These species are isostructural and cocrystallize with site disorder between Br and Cl in both halide positions, in an approximate 4:1 ratio of Br:Cl (Scheme 2).

**Scheme 2:**
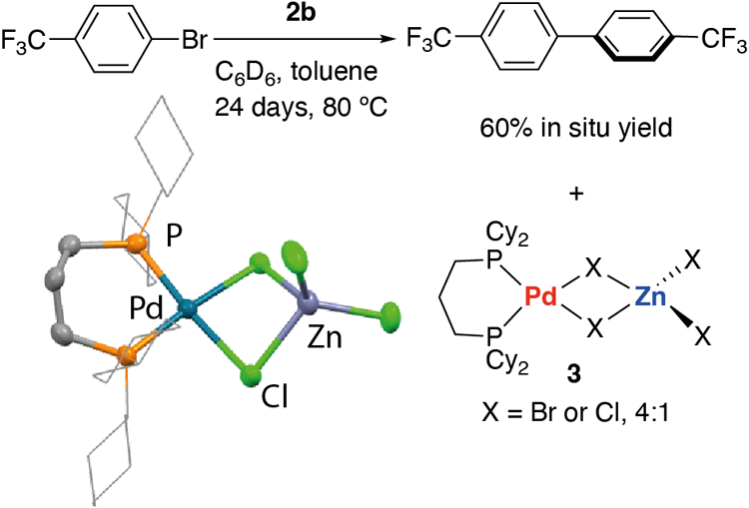
Reaction of **2b** with an Aryl Bromide The crystal structure of **3** is represented with chloride ligands.

In summary, we have isolated and crystallographically characterized two new Pd_2_Zn_2_ clusters supported by bis(phosphine) ligands formed by the reduction of palladium(II) precursors with metallic Zn. Analysis and calculations suggest that these are odd-electron species in the solid state, with the cluster held together by Pd–Pd and Pd–Zn interactions with limited evidence for Zn–Zn bonding. The formal oxidation state of Zn in these clusters is +1.5. As a result, they potentially represent arrested intermediates in the reduction process, in which Zn metal has been partially oxidized. Finally, these clusters show potential in cross-coupling reactions.

## Data Availability

Raw NMR data are available at the following repository: 10.14469/hpc/14998.

## Supporting Information Available

Experimental procedures, spectroscopic data, and computational details (PDF)
Calculated coordinates (XYZ)

## Supplementary Material

om4c00506_si_001.pdf

om4c00506_si_002.xyz
