# Supplementary material for: Flash Communication: Pd2Zn2 Clusters from the Reduction of Palladium(II) Dichloride Precursors with Metallic Zinc
Source: Organometallics. 2025 Mar 8;44(6):716–9. doi: 10.1021/acs.organomet.4c00506 (PMC11938339; doi:10.1021/acs.organomet.4c00506)
Supplement: Supplementary file 1 — om4c00506_si_001.pdf [file om4c00506_si_001.pdf]

Supporting Information

Flash Communication: Pd_2Zn_2 Clusters from the Reduction of Palladium(II) Dichloride Precursors with Metallic Zinc

Georgina Rai,[a] Martí Garçon,[a] Philip W. Miller,[a] Mark R. Crimmin[a]*

[a]Molecular Sciences Research Hub, Imperial College London, 82 Wood Lane, Shepherds Bush, London, W12 0BZ, UK.

m.crimmin@imperial.ac.uk

Raw NMR data are available at the following repository https://doi.org/10.14469/hpc/14998.

Table of Contents

General Experimental 3

Synthetic Procedures 4

NMR Spectroscopy 8

EPR Spectroscopy 14

X-ray Crystallography 15

Computational Methods 18

References 27

General Experimental

Unless otherwise stated, all manipulations were carried out using standard Schlenk and glovebox techniques, under inert atmosphere (dinitrogen or argon). An MBraun Labmaster glovebox was used, operating at <0.1 ppm H_2O and <0.1 ppm O_2. Solvents were dried over activated alumina from a solvent purification system (SPS) based upon the Grubbs design and degassed before use. Glassware and PTFE stirrer bars were dried for >6 h prior to use at 120 °C. Benzene-d_6 was degassed by freeze-pump-thaw cycles and stored over 3 Å molecular sieves before use. All reagents were acquired from commercial suppliers (Sigma Aldrich, VWR, Fluorochem, Apollo Scientific) and used without further purification unless specified. [$PdCl_2(NCPh)_2$], **1a** and **1b** were prepared according to literature procedures.[1,2] Zn dust was acquired from Sigma Aldrich and activated prior to use by washing with HCl, water, acetone, and diethyl ether consecutively according to a literature procedure.[3]

^1H, $^{31}P\{^1H\}$ and ^{19}F NMR spectra were recorded in J. Young's NMR tubes on BRUKER 400 MHz or 500 MHz spectrometers. ^1H NMR chemical shifts (δ) were referenced to internal solvent resonances, while $^{31}P\{^1H\}$ and ^{19}F NMR chemical shifts were referenced to 85% H_3PO_4 and $CFCl_3$ respectively. Data were processed using the MestReNova software package. The coupling constants (*J*) are reported in Hertz (Hz). The following abbreviations are used to define multiplicities: s (singlet), d (doublet), t (triplet), q (quartet), m (multiplet), br s (broad signal). ^{19}F NMR spectroscopic yields were calculated using benzene-d_6 solutions of hexafluorobenzene inserts, with d_1 = 55 s acquisition parameters.

Single crystal X-ray data were collected using an Agilent Xcalibur PX Ultra A diffractometer, and the structures were refined using the OLEX2 and SHELX-2019 program systems.[4-6] Details of the individual data collection are provided in the relevant CIFs. A summary of the fundamental crystal and refinement data are given in Table S1. Atomic coordinates, anisotropic displacement parameters and bond lengths and angles can be found in the .cif files, which have been deposited in the Cambridge Crystallographic Data Centre.

EPR experiments were performed in the PEPR-Centre for Pulse EPR Spectroscopy at Imperial College London using a Bruker Elexsys E580 spectrometer.

Synthetic Procedures

Synthesis of **2a**

[Pd(dcpe)Cl_2] (100 mg, 0.17 mmol) and activated Zn dust (43.6 mg, 0.67 mmol) were suspended in dry THF (10 mL). The mixture was stirred at 80 °C for 72 h. The cooled solution was filtered through celite, and evaporated to dryness. The residue was dissolved in toluene (3 mL), and subsequently filtered through glass fibre to remove solid by-products. The solution was concentrated and layered with n-hexane (3 mL). The yellow, cloudy n-hexane layer was extracted into a separate vial. This process was repeated three times. The combined extractions were concentrated down, and minimal toluene added until all solid had solubilised. The solution was then left at -35 °C, affording yellow needle-shaped crystals and a yellow amorphous solid. The yellow amorphous solid could be washed away quickly with toluene.

^{1}H NMR (400 MHz, $CDCl_3$) δ (ppm): 1.27–2.05 (uncharacteristic series of overlapping m). Paramagnetic ^{1}H NMR (sweep width 600 ppm) displayed no extra peaks.

^{31}P{^{1}H} NMR (162 MHz, $CDCl_3$) δ (ppm): -14.08 (s).

^{13}C NMR (126 MHz, $CDCl_3$) δ (ppm): 31.7 (t, $^1J_{C\text{-}P}$ = 8.2 Hz, *ipso*-Ar-**C**), 28.8 (s, *para* or *meta*-Ar-**C**), 28.1 (s, *para* or *meta*-Ar-**C**), 27.1 (t, $^2J_{C\text{-}P}$ = 5.4 Hz, *ortho*-Ar-**C**), 27.0 (t, $^2J_{C\text{-}P}$ = 6.2 Hz, *ortho*-Ar-**C**), 25.8 (s, *para* or *meta*-Ar-**C**), 15.7 (t, $^1J_{C\text{-}P}$ = 10.8 Hz, P-**C**H_2).

HRMS (ESI): *m/z* calculated for $C_{52}H_{96}Cl_2P_4Pd_2Zn_2$ (M – Cl)$^+$ 1258.2465, not found.

IR (ATR, cm^{-1}), v: 2922 (C–H stretch), 2848 (C–H stretch), 1446 (–CH_2– bend).

Synthesis of **2b**

[Pd(dcpp)Cl_2] (50 mg, 0.081 mmol) and activated Zn dust (21.3 mg, 0.33 mmol) were suspended in dry THF (5 mL). The mixture was stirred at 80 °C for 24 h. The cooled solution was filtered through celite, and evaporated to dryness. The residue was dissolved in toluene (1.5 mL) and n-hexane (0.5 mL) and left at -35 °C for 3 h. The resulting supernatant was removed from an undesired brown oil, transferred to a separate vial, and stored at -35 °C overnight. This afforded orange crystals, brown oil and a yellow amorphous solid. The brown oil and yellow amorphous solid could be washed away quickly with toluene affording orange crystals (3.5 mg, 2.6 µmol, 6%).

^{1}H NMR (400 MHz, $CDCl_3$) δ (ppm): 1.27–2.05 (uncharacteristic series of overlapping m). Paramagnetic ^{1}H NMR (sweep width 600 ppm) displayed no extra peaks.

^{31}P{^{1}H} NMR (162 MHz, $CDCl_3$) δ (ppm): -10.68 (s).

^{13}C NMR (101 MHz, $CDCl_3$) δ (ppm): 32.6 (t, $^1J_{C\text{-}P}$ = 8.8 Hz, *ipso*-Ar-**C**), 28.5 (s, *para* or *meta*-Ar-**C**), 27.7 (s, *para* or *meta*-Ar-**C**), 27.2 (t, $^2J_{C\text{-}P}$ = 5.2 Hz, *ortho*-Ar-**C**), 27.2 (t, $^2J_{C\text{-}P}$ = 5.7 Hz, *ortho*-Ar-**C**), 26.0 (s, *para* or *meta*-Ar-**C**), 20.1 (2, CH_2-**C**H-CH_2), 19.4 (t, $^1J_{C\text{-}P}$ = 4.5 Hz, P-**C**H_2)

HRMS (ESI): *m/z* calculated for $C_{54}H_{100}Cl_2P_4Pd_2Zn_2$ (M – Cl)$^+$ 1286.2778, found 1289.2822.

IR (ATR, cm^{-1}), v: 2922 (C–H stretch), 2848 (C–H stretch), 1444 (–CH_2– bend).

IR data for both **2a** and **2b** are consistent with both the dcpe and dcpp ligand environments.

Due to the lack of pure samples on a multi-milligram scale, CHN analyses could not be carried out.

Synthesis of 4,4'-bis(trifluoromethyl)biphenyl and [Pd(dcpp)X_4Zn] (**3**, X = Br, Cl)

2b (3.5 mg, 2.6 µmol) and 4-bromobenzotrifluoride (0.53 M solution in C_6D_6, 10 µL, 5.3 µmol, 2 equiv.) were dissolved in C_6D_6 (0.5 mL) and the solution transferred to a J. Young's NMR tube, with a hexafluorobenzene internal standard capillary insert. The amount of homocoupled product formed was monitored by ^{19}F and ^{1}H NMR spectroscopy over a period of 24 days, until 100% conversion and 60% yield of homocoupled product was achieved, along with 34% of α,α,α-trifluorotoluene side product. Colourless crystals of **3** suitable for SCXRD were deposited on the side of the NMR tube.

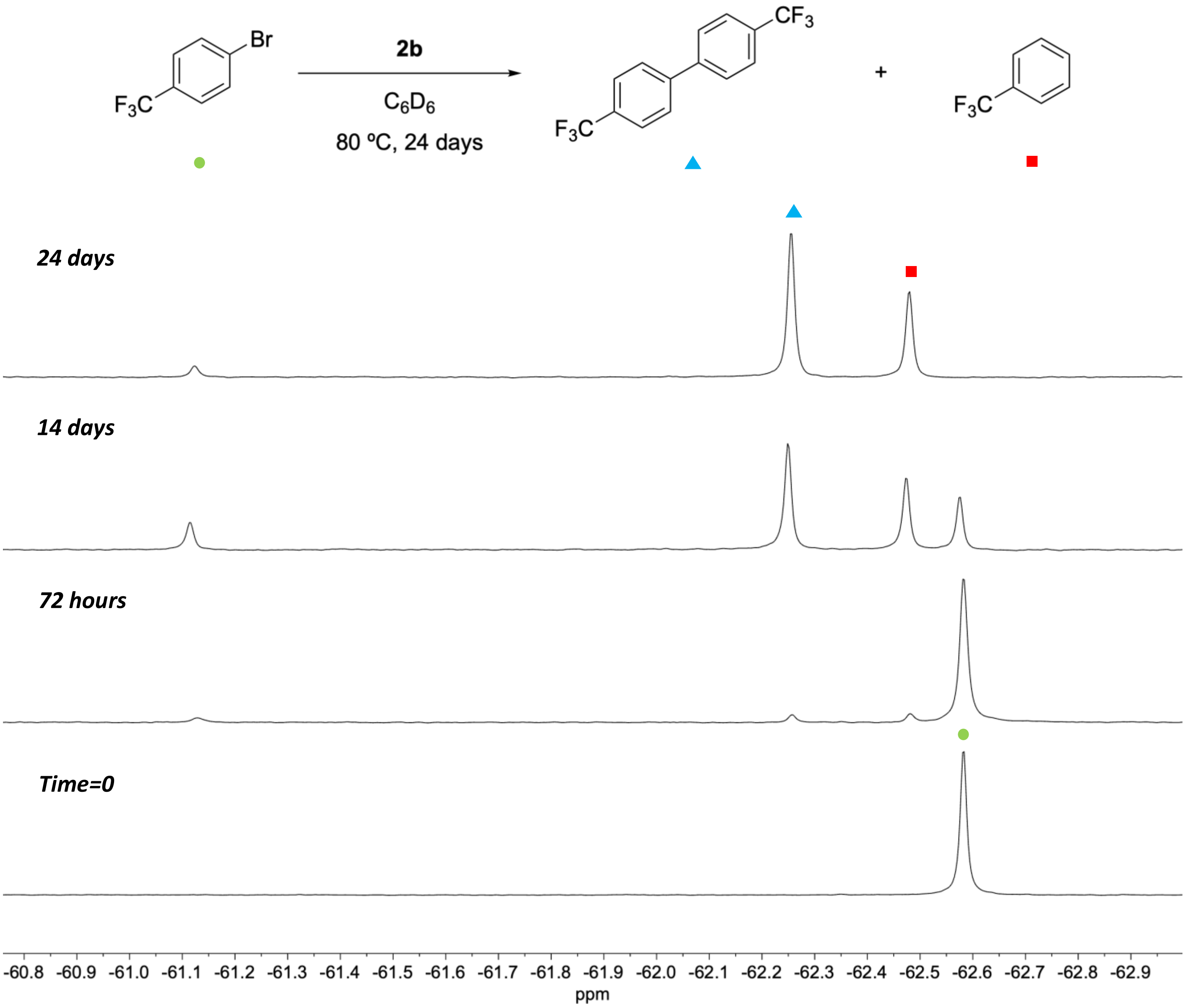

Figure S1. ^{19}F NMR spectra for the time-course of the homocoupling reaction mediated by complex **2b**.

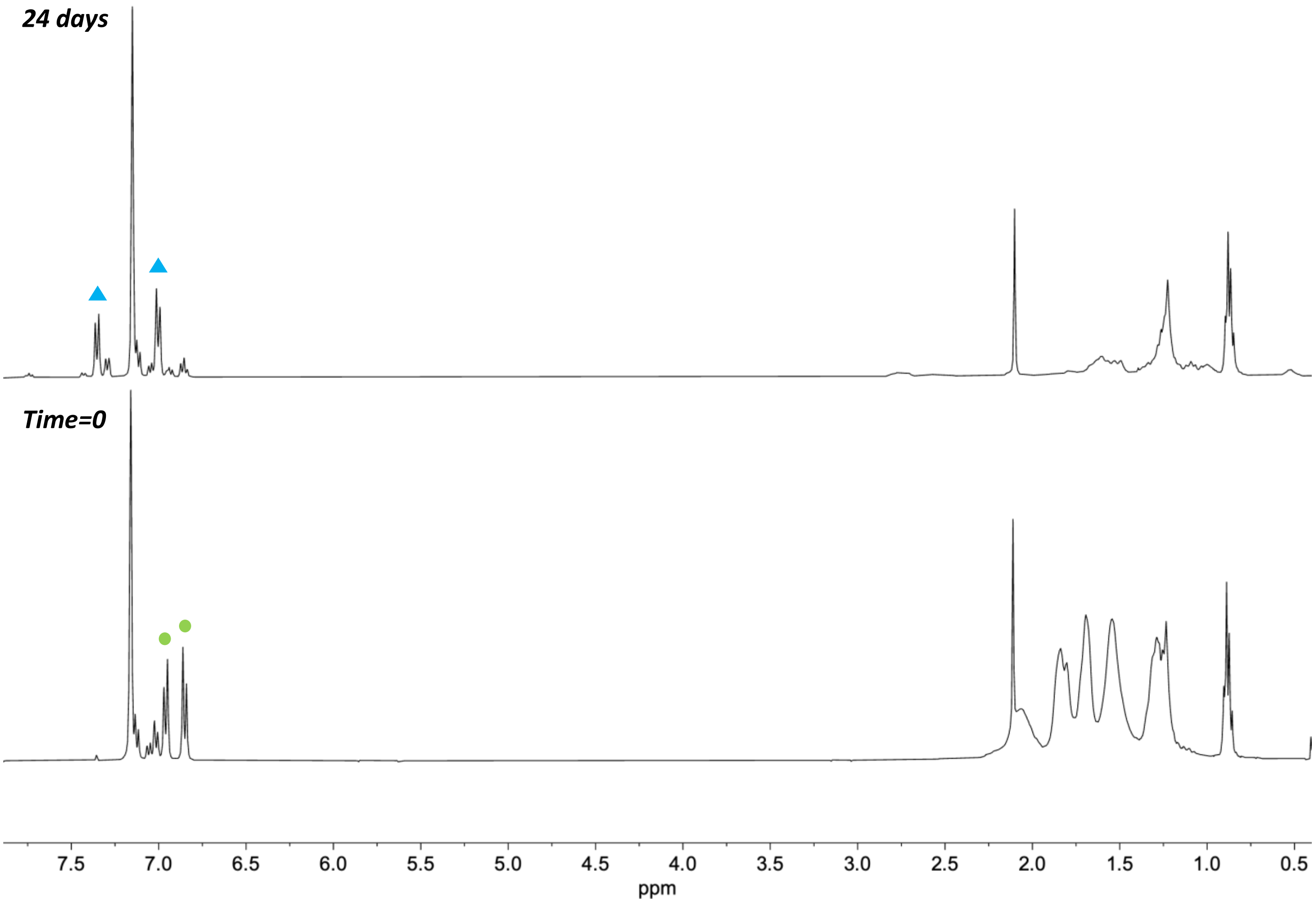

Figure S2. ^{1}H NMR spectra for before (bottom) and after (top) the homocoupling reaction mediated by complex **2b**.

To confirm the identity of the hydrodebrominated side product, a sample of α,α,α-trifluorotoluene was added to the reaction mixture and the associated peaks increased in size.

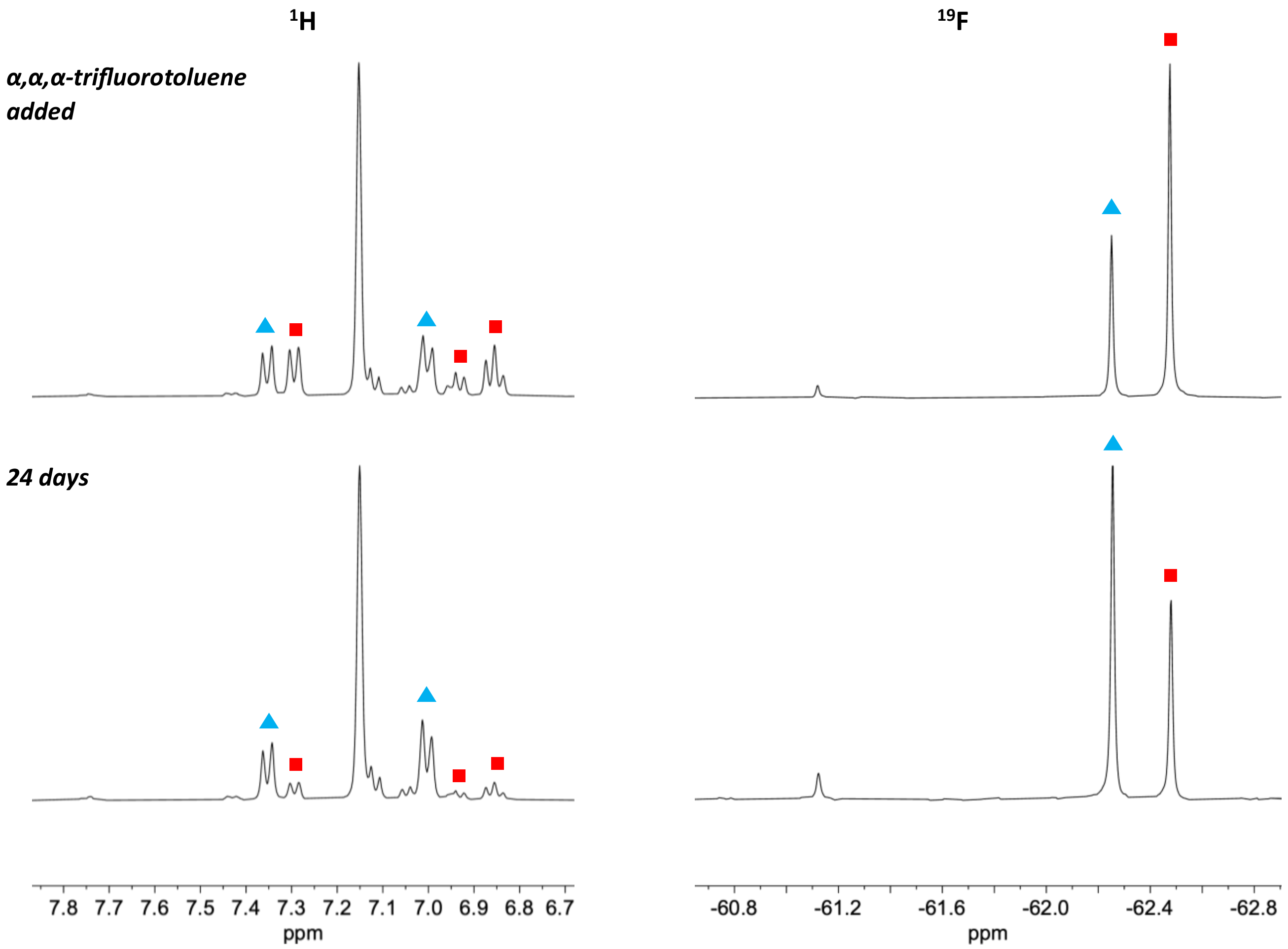

Figure S3. Left: ^{1}H NMR spectra of the final reaction mixture (bottom) and with extra α,α,α-trifluorotoluene added (top). Right: ^{19}F spectra of the final reaction mixture (bottom) and with extra α,α,α-trifluorotoluene added (top).

NMR Spectroscopy

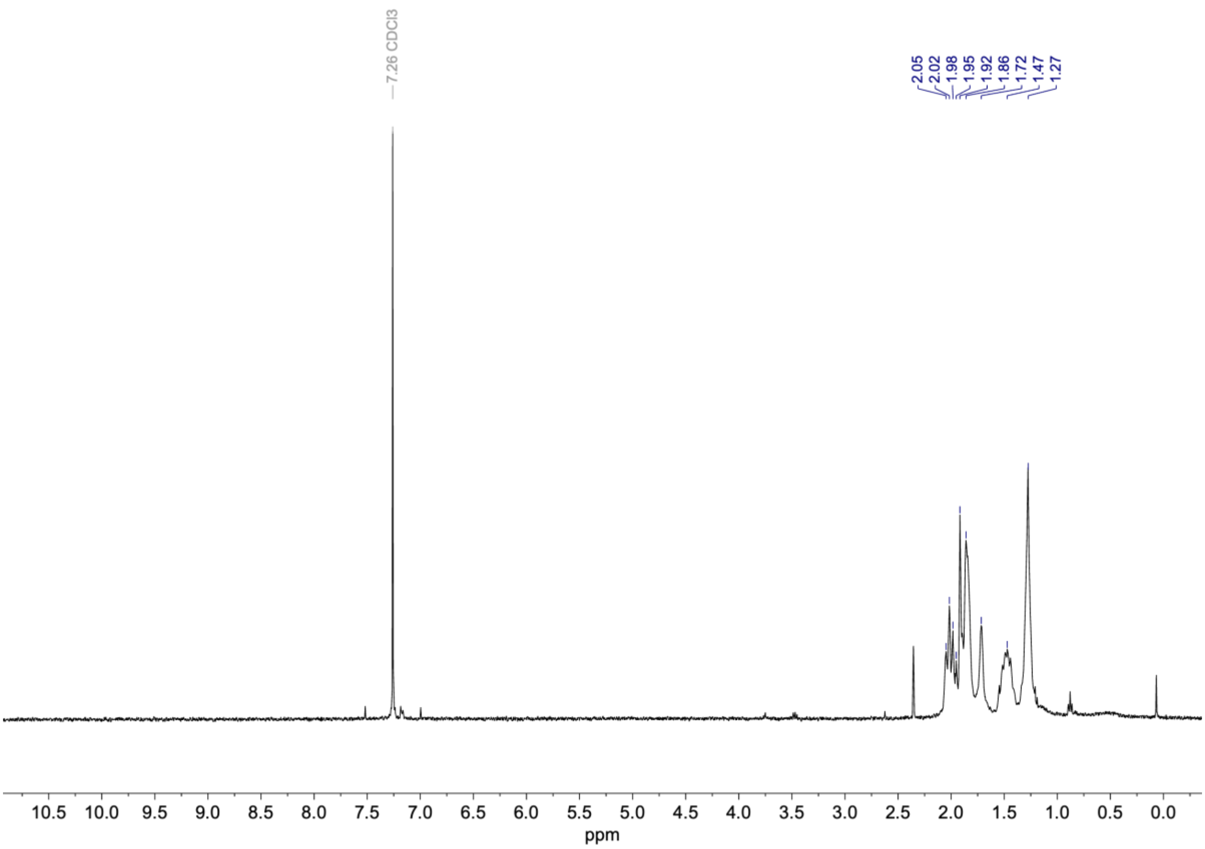

Figure S4. ^{1}H NMR spectrum of **2a** (400 MHz, $CDCl_3$, 298 K).

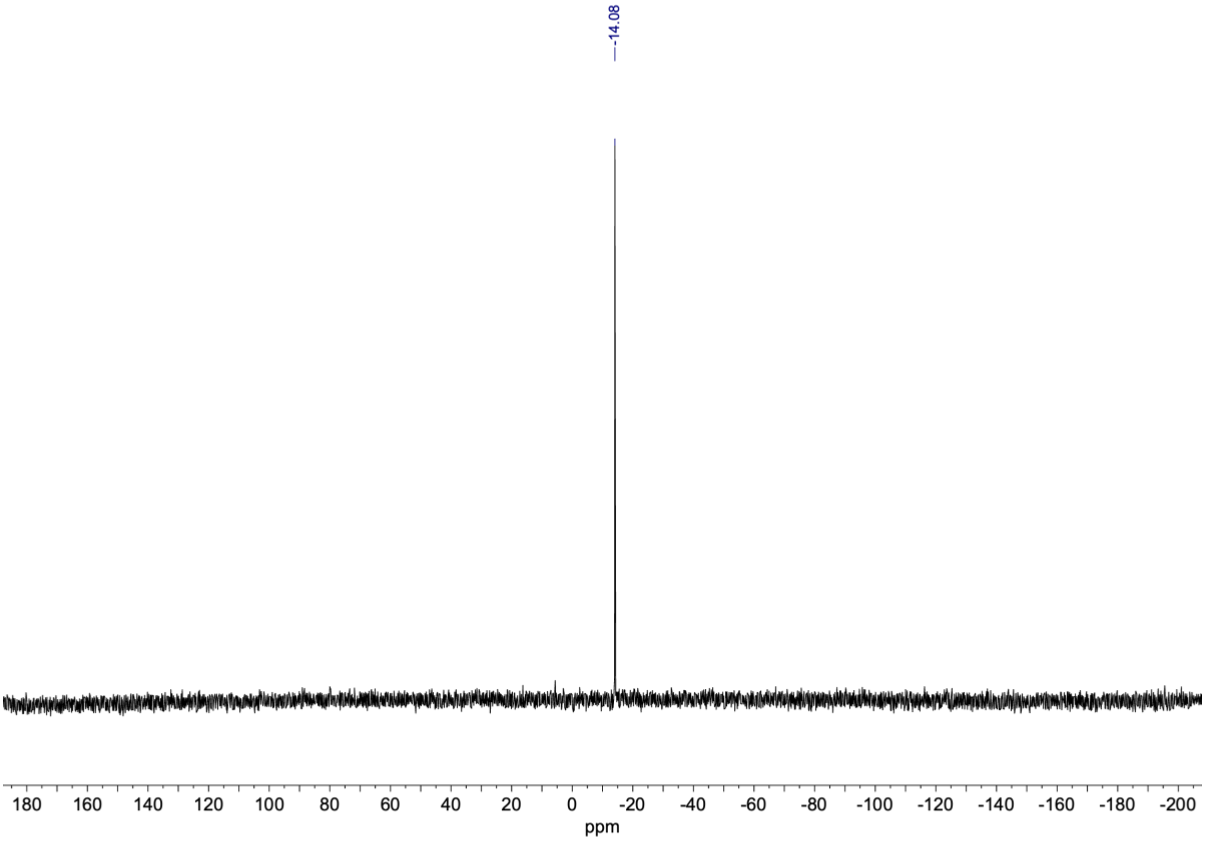

Figure S5. ^{31}P NMR spectrum of **2a** (400 MHz, $CDCl_3$, 298 K).

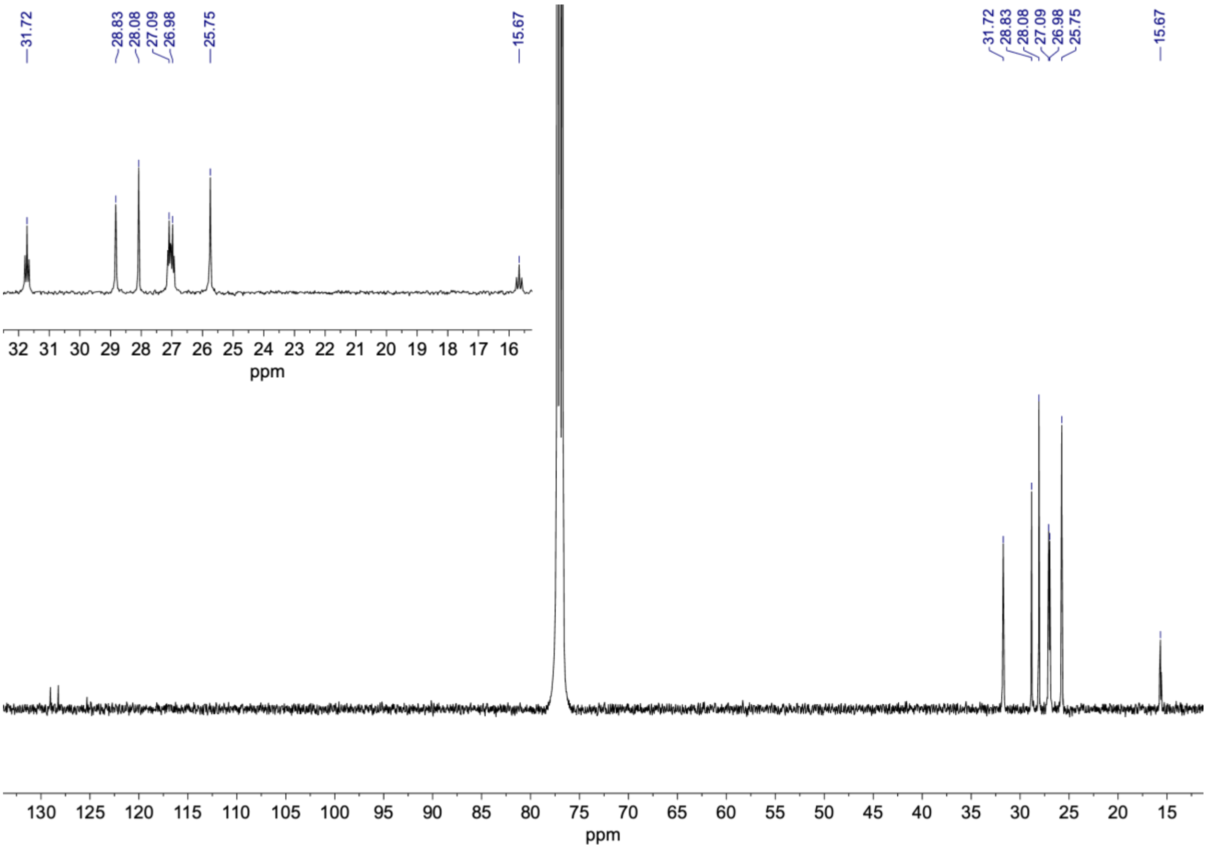

Figure S6. ^{13}C NMR spectrum of **2a** (500 MHz, $CDCl_3$, 298 K).

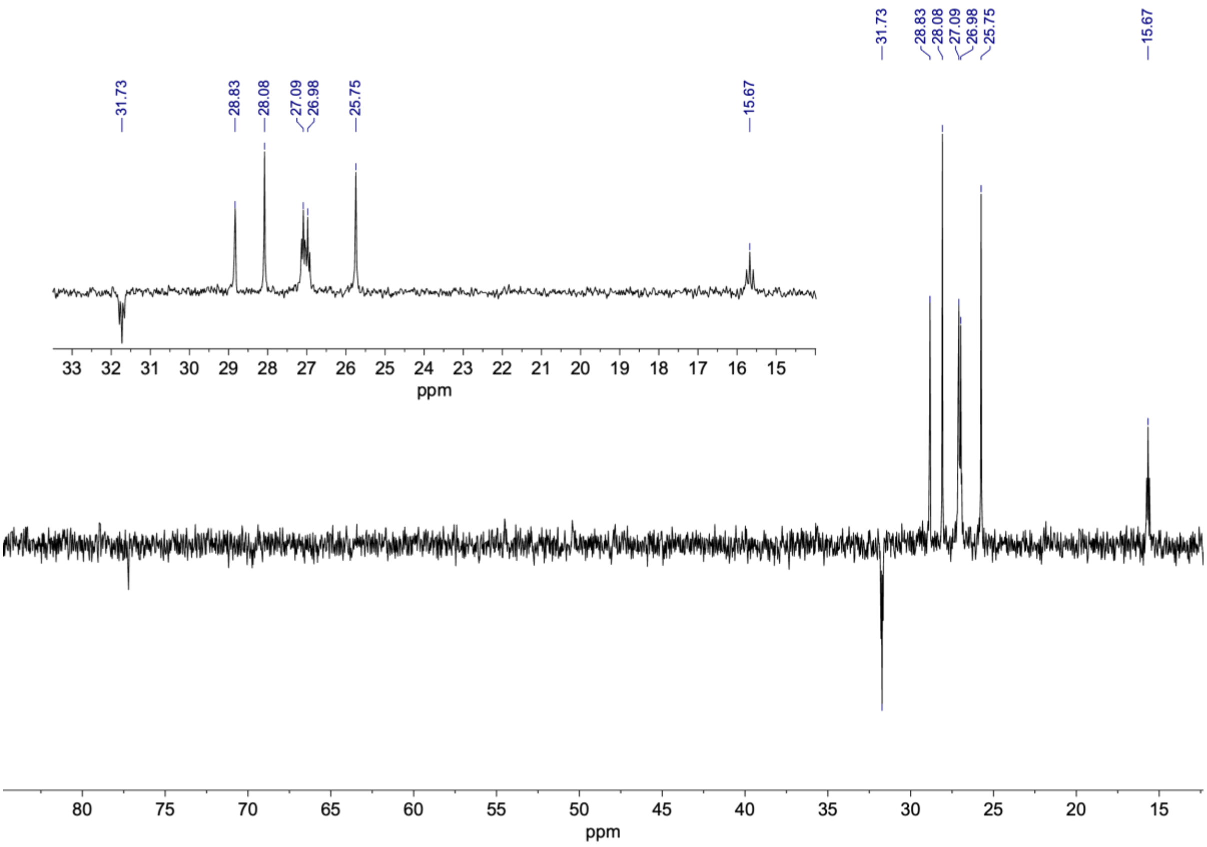

Figure S7. DEPT-135 ^{13}C NMR spectrum of **2a** (500 MHz, $CDCl_3$, 298 K).

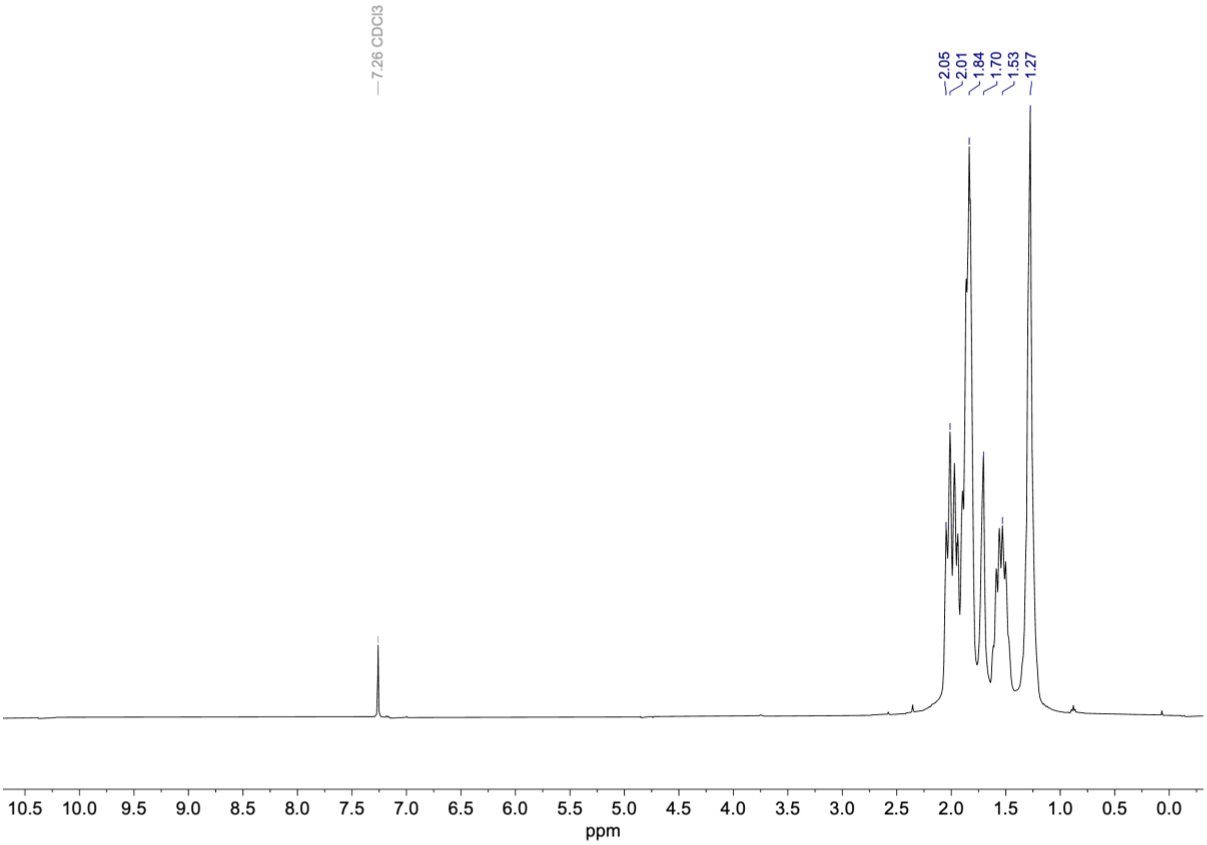

Figure S8. ^{1}H NMR spectrum of **2b** (400 MHz, $CDCl_3$, 298 K).

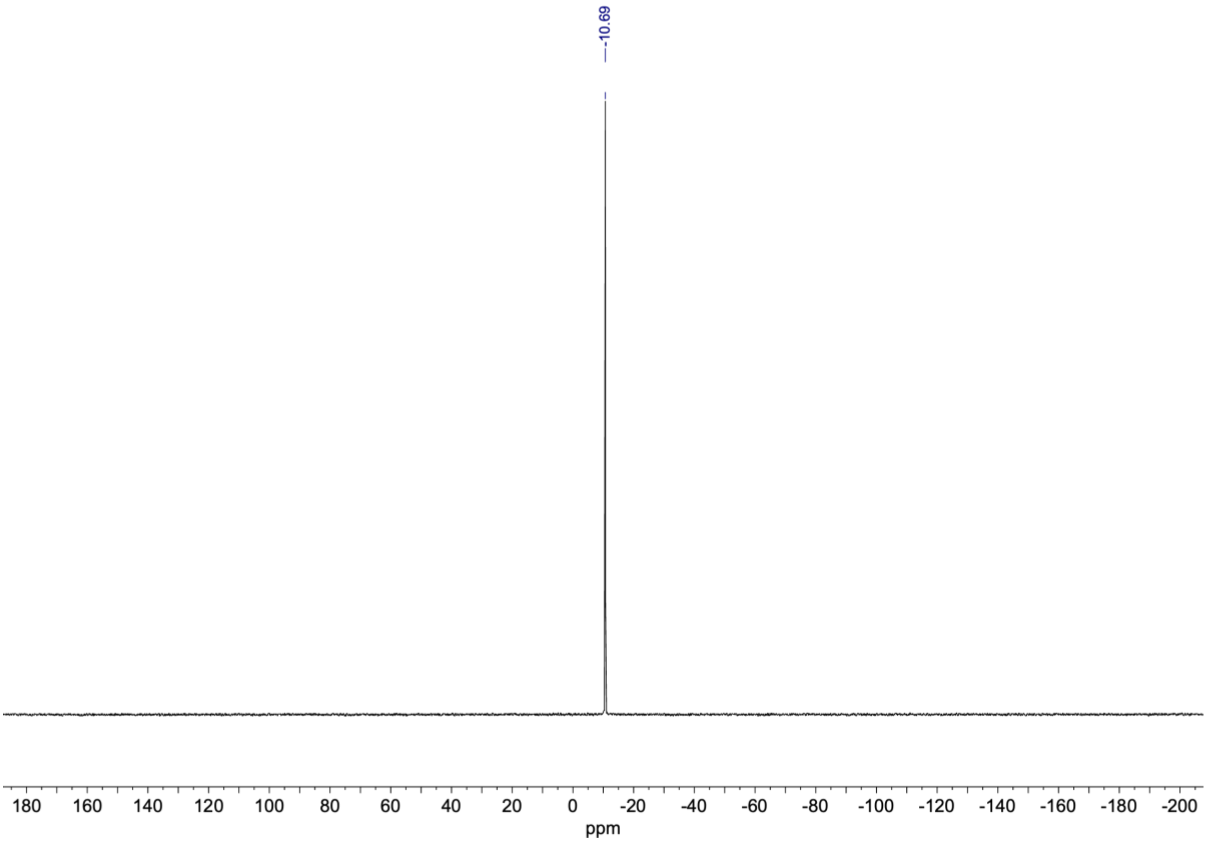

Figure S9. ^{31}P NMR spectrum of **2b** (400 MHz, $CDCl_3$, 298 K).

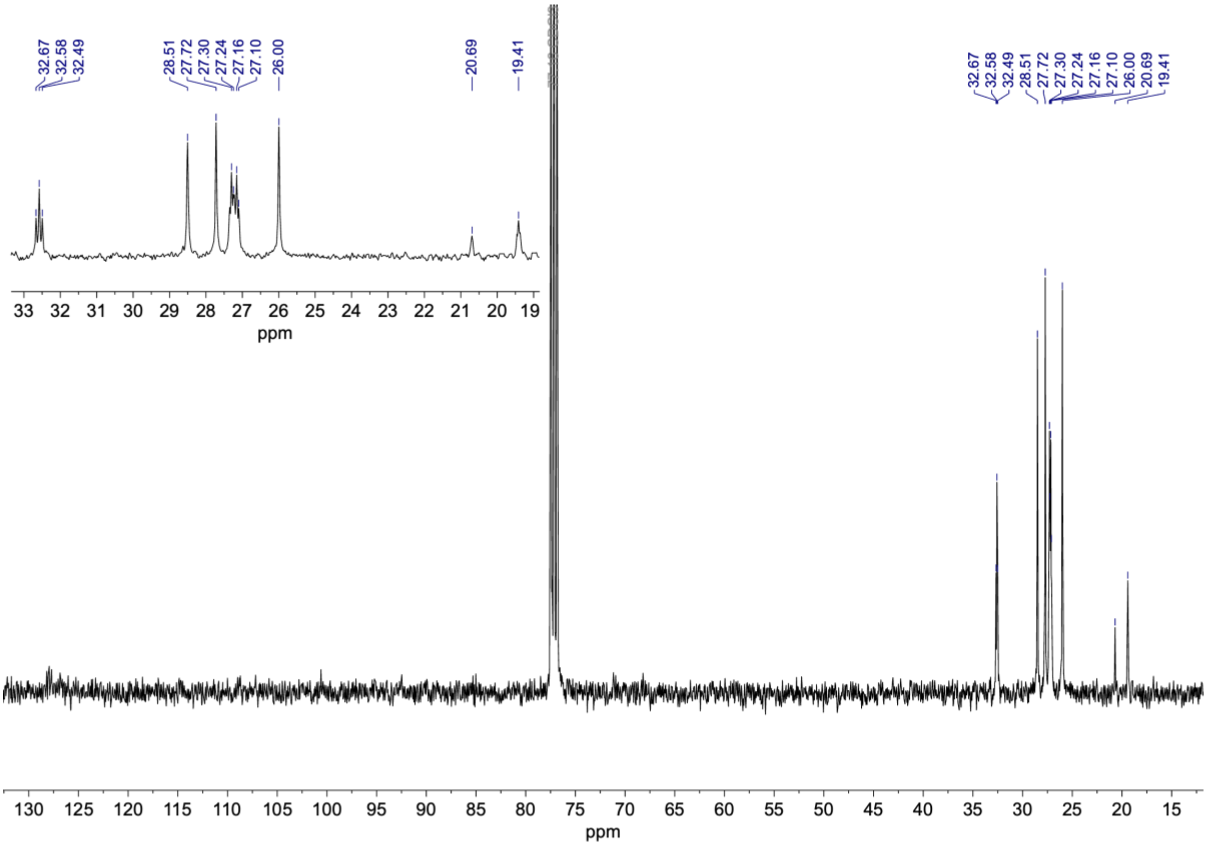

Figure S10. ^{13}C NMR spectrum of **2b** (400 MHz, $CDCl_3$, 298 K).

IR Spectroscopy

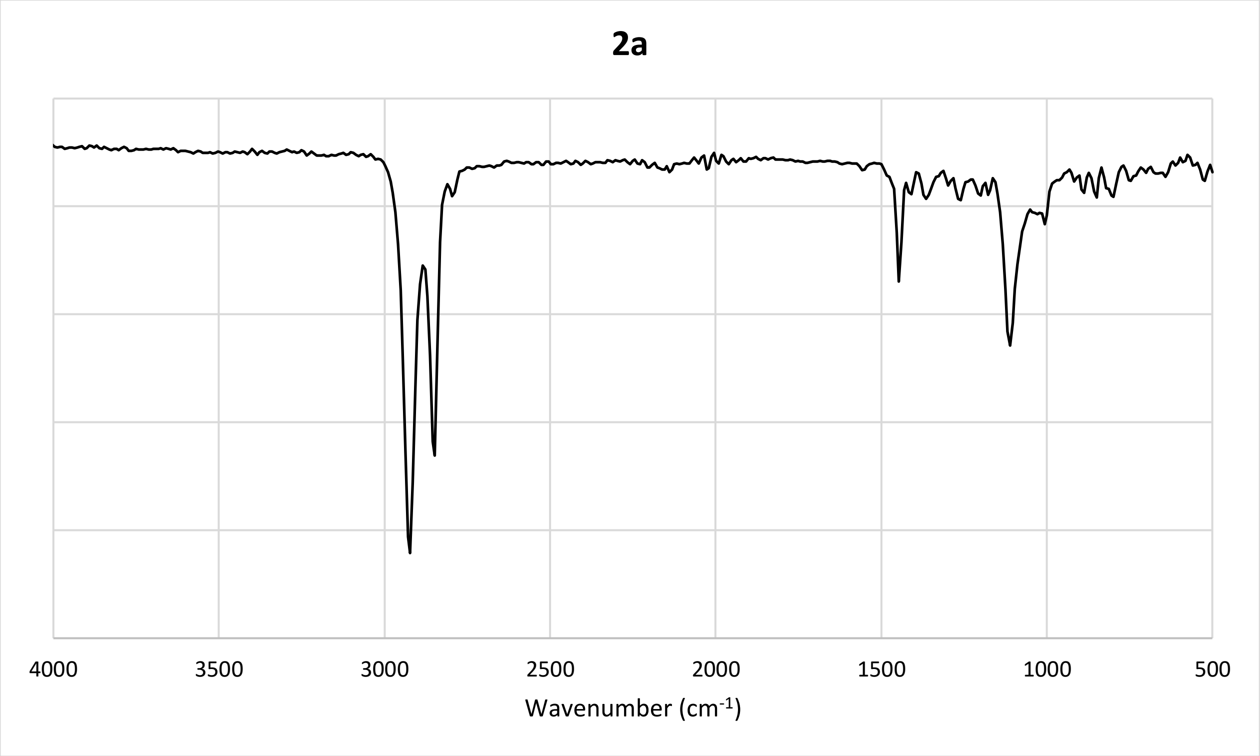

Figure S11. Infrared spectrum of **2a**. Recorded on AT-IR as a film from THF solution.

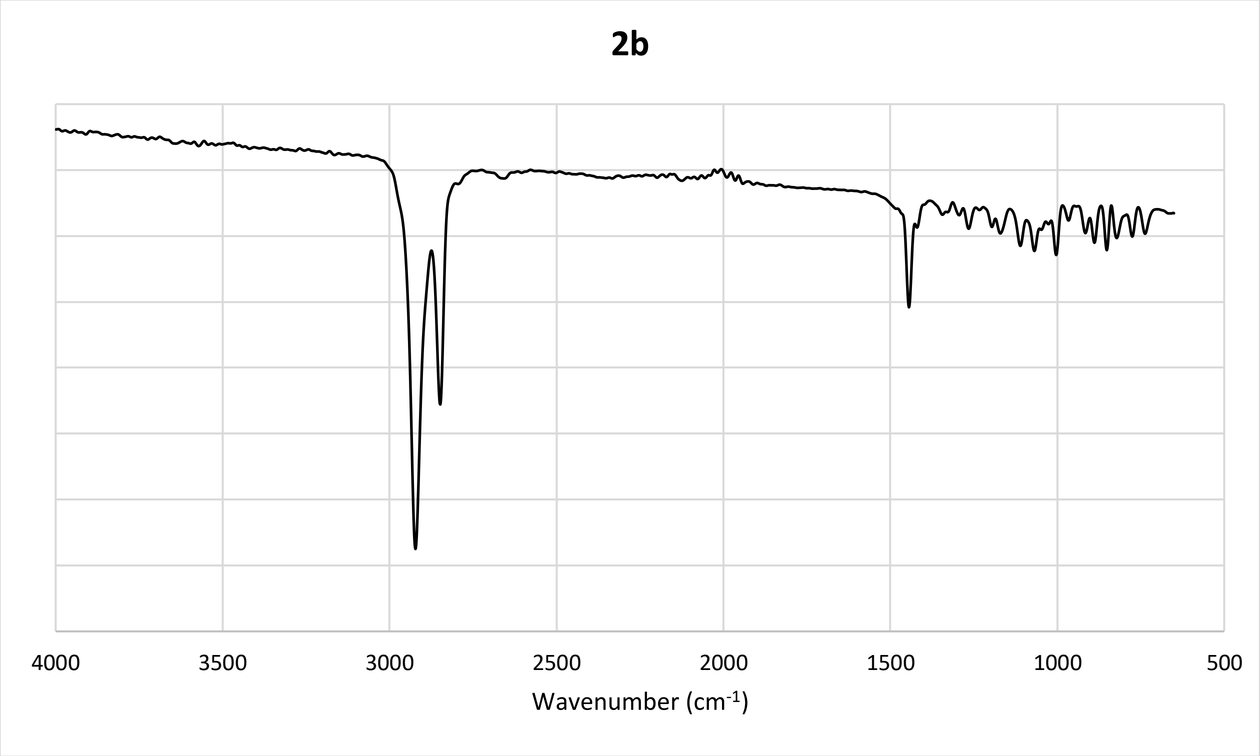

Figure S12. Infrared spectrum of **2b**. Recorded on AT-IR as a film from THF solution.

Mass Spectrometry

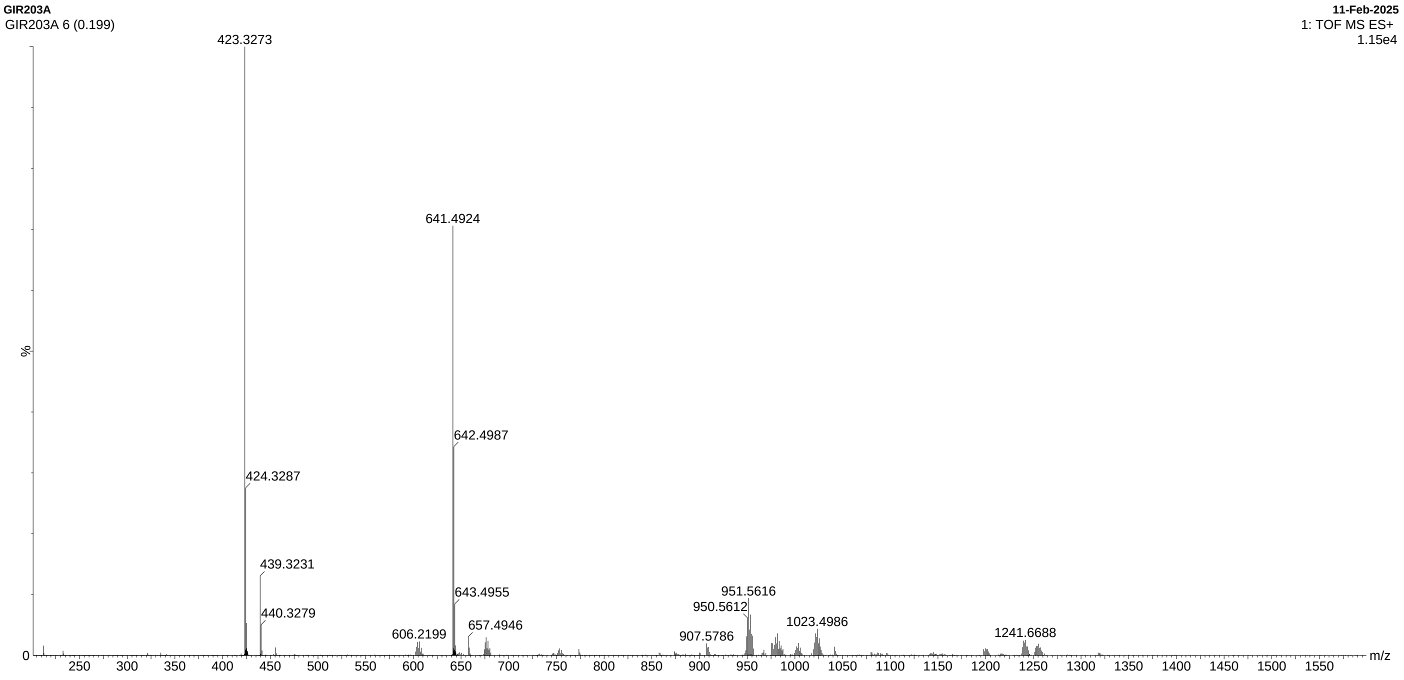

Figure S13. Mass spectrum of **2a**.

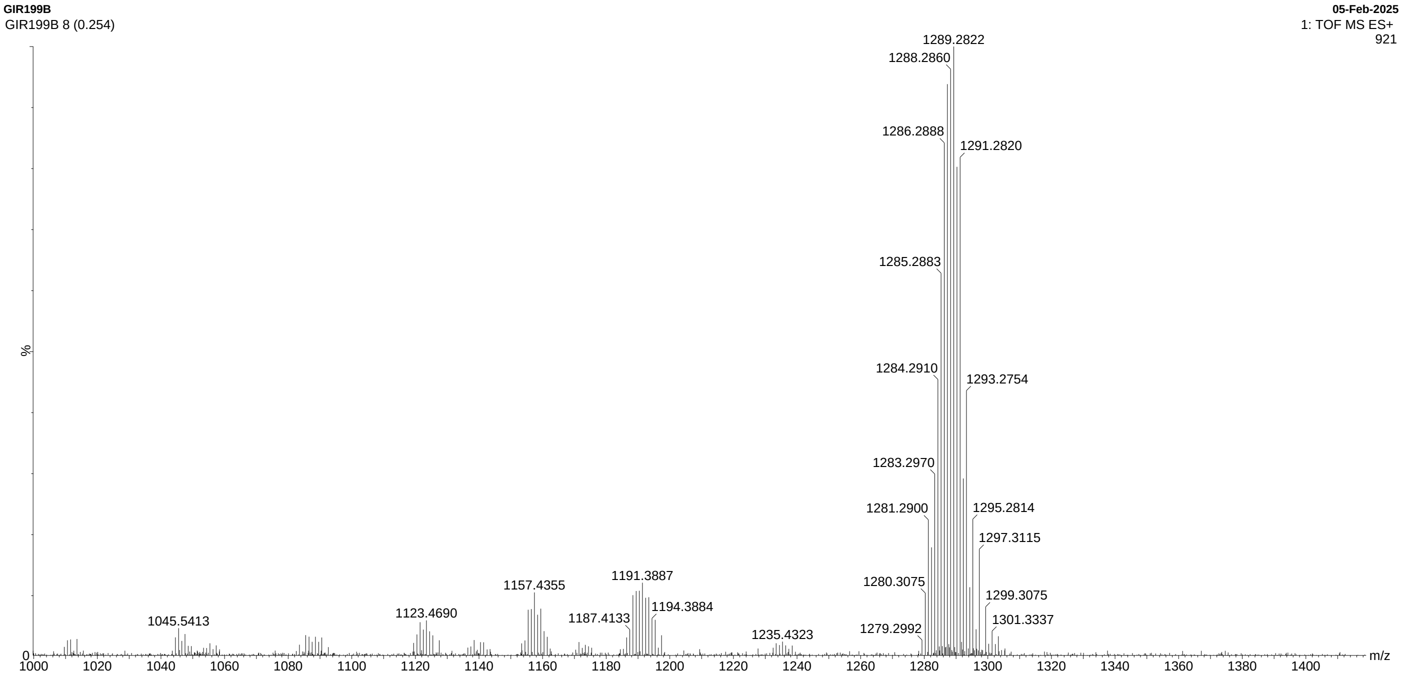

Figure S14. Mass spectrum of **2b**, zoomed in on the (M – Cl)$^{+}$ peak.

EPR Spectroscopy

Sample preparation and data acquisition

A sample of **2b** for EPR characterisation was prepared by dissolving the complex in 2:1 toluene:$CDCl_3$ and loading the solution in a 3.8 mm OD quartz tube fitted with a J. Young's style stopcock. The magnetic field of the EPR spectrometer was calibrated using DPPH as a standard (g = 2.0036).[7] The frozen-matrix CW-EPR spectrum was recorded on a Bruker Elexsys E580 spectrometer at a temperature of 40 K using a microwave power of 8.0 mW, a field modulation amplitude of 20 G, field modulation frequency of 100 kHz, a conversion time of 40.96 ms, a time constant of 10.24 ms and a magnetic field sweep rate of 29.8 Gs^{-1}. All data analysis was carried out using EasySpin.[8]

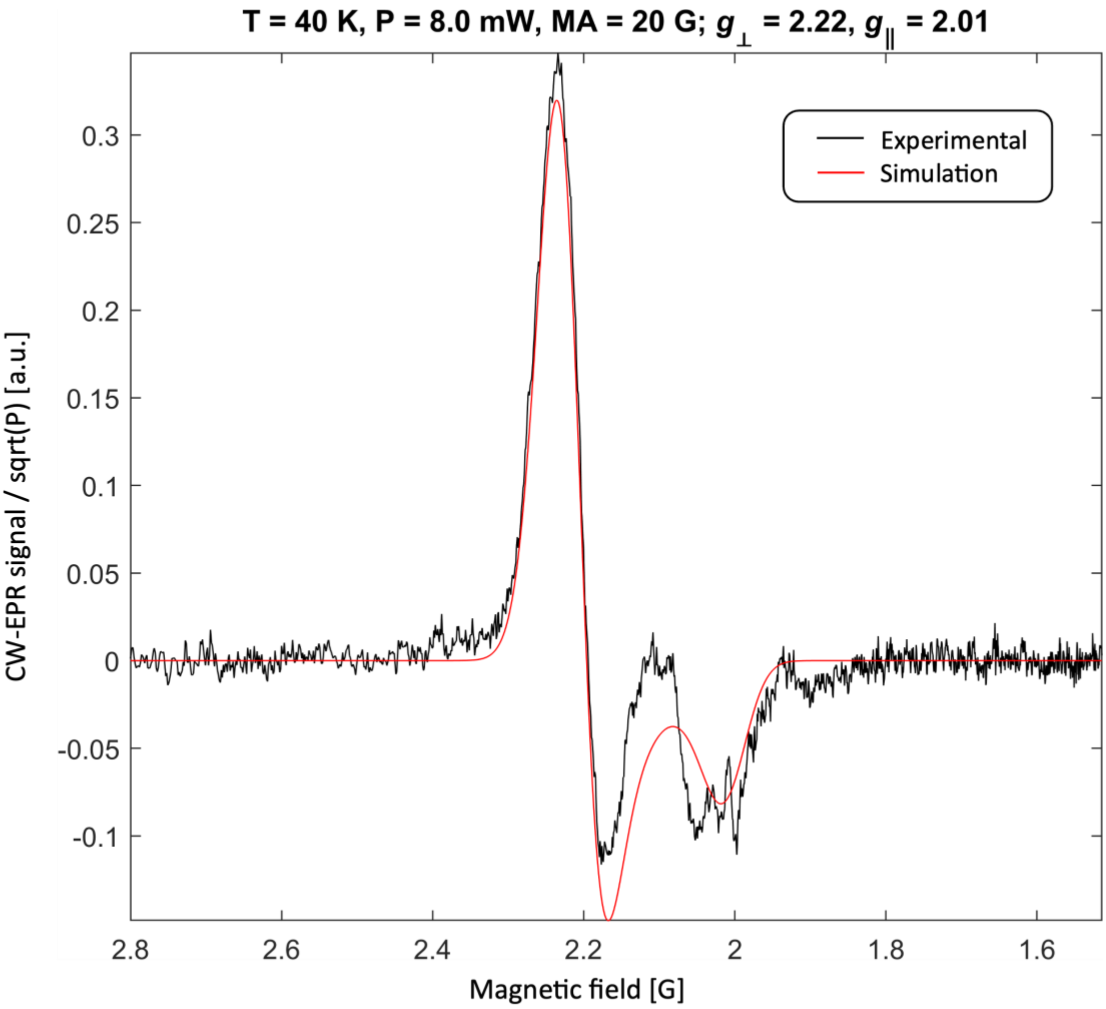

Figure S15. X Band CW EPR of **2b** in 2:1 toluene:$CDCl_3$ (40 K). Experimental data (black) and simulated spectra (red).

X-ray Crystallography

X-ray data

Table S1. Summary of the crystallographic data for the structures of **2a**, **2b** and **3**. Data were collected using an Xcalibur PX Ultra A (**2b**) diffractometer, and the structures were solved and refined using the OLEX2[4] and SHELX-2019[5,6] program systems. [a]Completeness to 0.84 Å resolution. [b]$R_1 = \Sigma||F_o| - |F_c||/\Sigma|F_o|$; $wR_2 = \{\Sigma[w(F_o^2 - F_c^2)^2] / \Sigma[w(F_o^2)^2]\}^{1/2}$; $w^{-1} = \sigma_2(F_o^2) + (aP)^2 + bP$. [c]The asymmetric unit contains two independent molecules.

**compound**	**2a**	**2b**	**3**
**CCDC No.**	2393999	2394000	2394001
**formula**	$C_{52}H_{96}Cl_3P_4Pd_2Zn_2$	$C_{54}H_{100}Cl_3P_4Pd_2Zn_2$	$C_{27}H_{50}Br_{3.124}Cl_{0.876}P_2PdZn$
**solvent**	$0.5(C_7H_8)$	$1.5(C_7H_8)$, $0.5(C_5H_{12})$	$0.5(C_6H_6)$
**formula weight**	1341.12	1497.38	925.06
**colour, habit**	yellow needle	yellow block	colourless block
**temperature / K**	173	173	173
**crystal system**	monoclinic	triclinic	triclinic
**space group**	*P*2₁/*n* (no. 14)	*P*–1 (no. 2)	*P*–1 (no. 2)
a **/ Å**	11.8803(2)	12.0301(7)	10.5875(3)
b **/ Å**	14.2380(2)	12.8331(7)	18.0703(8)
c **/ Å**	36.3463(5)	23.7366(8)	19.8973(10)
**α / deg**	90	86.854(4)	85.641(4)
**β / deg**	91.3300(10)	81.702(4)	82.306(3)
**γ / deg**	90	77.113(5)	73.202(3)
V **/ Å³**	6146.38(16)	3533.9(3)	3608.7(3)
Z	4	2	4[c]
Dc **/ g cm⁻³**	1.449	1.407	1.703
**radiation used**	Cu-Kα	Cu-Kα	Cu-Kα
**μ / mm⁻¹**	10.411	6.979	10.411
**no. of unique reflections**			
**measured (** R_{int} **)**	11804 (0.0401)	13450 (0.0588)	13834 (0.0405)
**obs, |*Fo*|>4σ(|*Fo*|)**	9357	10090	10754
**completeness (%)**[a]	98.7	98.3	98.7
**no. of variables**	632	788	723
R_1 **(obs),** wR_2 **(all)**[b]	0.03920, 0.0973	0.0574, 0.1632	0.0430, 0.1167

Refinement details

2a: The complex was found to crystallize with 0.5 equivalents of toluene solvent, disordered across a centre of inversion located ca. 1.44 Å away from the ***C***–CH_3 atom. This was modelled in one unique orientation with the non-hydrogen atoms being anisotropic (and its symmetry related orientation). The disordered atoms were restrained to be similar.

2b: The complex was found to crystallize with three solvent molecules which were identified as 1.5 equivalent of toluene and 0.5 equivalents of pentane. The first toluene molecule was found to be disordered. It was modelled across two unique orientations at a ratio of ca. 0.63:0.37. Only the non-hydrogen atoms of the major orientation were modelled anisotropically, the rest were modelled isotropically. The second toluene molecule was found to be disordered across a centre of inversion located ca. 0.769 Å away from the ***C***–CH_3 atom. This was modelled in one unique orientation with the non-hydrogen atoms being anisotropic (and its symmetry related orientation). The pentane molecule was found to be disordered across a centre of inversion located ca. 0.34 Å away from the central carbon (in the major orientation). It was modelled in two unique orientations at a ratio of ca. 0.29:0.21 (and their symmetry related orientations). For both orientations, all atoms were modelled isotropically. For all the above disorders, the disordered orientations were restrained to be similar.

3: The asymmetric unit contains two independent molecules of **3** and 1 molecule of included benzene solvent (0.5 equivalent per molecule of **3**). The C10-based cyclohexyl group was found to be disordered. It was modelled in two orientations at a ratio of ca. 0.84:0.16. The C1A-based propyl group was found to be disordered. Only the central methylene was modelled in two orientations at ratio of ca. 0.88:0.12, ie. sharing the same P***C***H_2 groups. For both of the above disorders the two orientations were restrained to be similar and only the non-hydrogen atoms of the major orientation were modelled anisotropically, the rest were modelled isotopically. The included benzene solvent molecule was modelled in one unique orientation with all non-hydrogen atoms anisotropic. All 8 unique Br atoms were found to exhibit same site disorder between Br/Cl. All Br/Cl atoms were refined anisotropically. The only acceptable model was obtained by letting each Br/Cl ratio refine independently using their own free variable, using same cartesian coordinate (EXYZ) and same anisotropic displacement (EADP) constraints. This yielded Br/Cl ratios of ca. Br1/Cl1: 0.71:0.29, Br2/Cl2: 0.82:0.18, Br3/Cl3: 0.74:0.26, Br4/Cl4: 0.72:0.28, Br1A/Cl1A: 0.73:0.27, Br2A/Cl2A: 0.77:0.23, Br3A/Cl3A: 0.76:0.24 and Br4A/Cl4A: 0.87:0.13, giving the average Br/Cl ratio of ca. 0.78:0.22.

Crystal structures

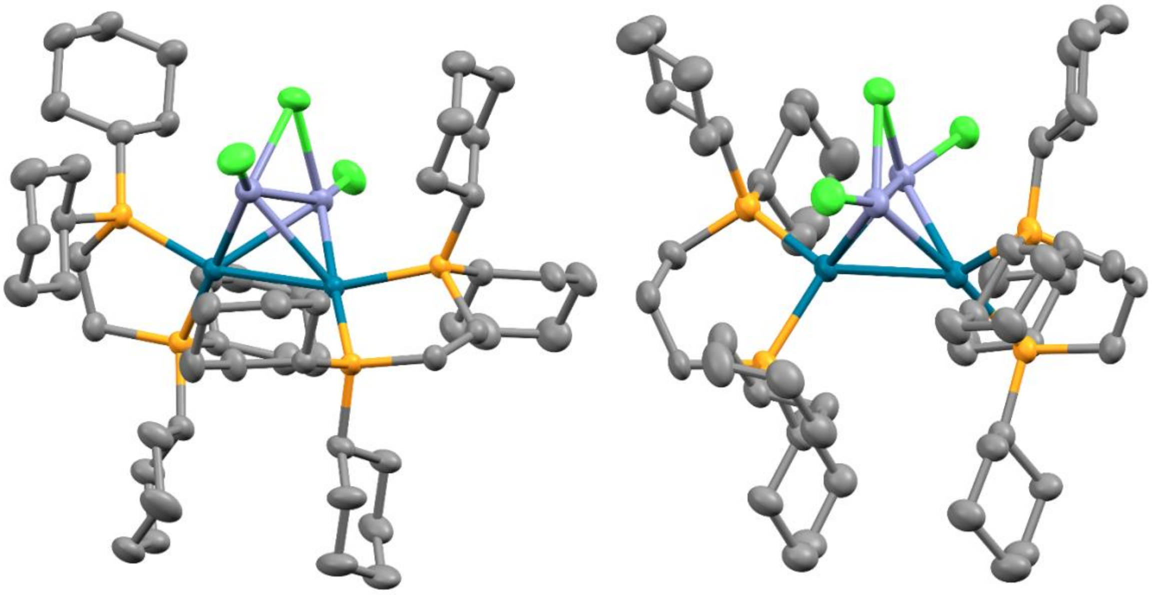

Figure S16. Crystal structure of left: **2a** and right: **2b**. All hydrogen atoms and the included solvent molecules are omitted for clarity.

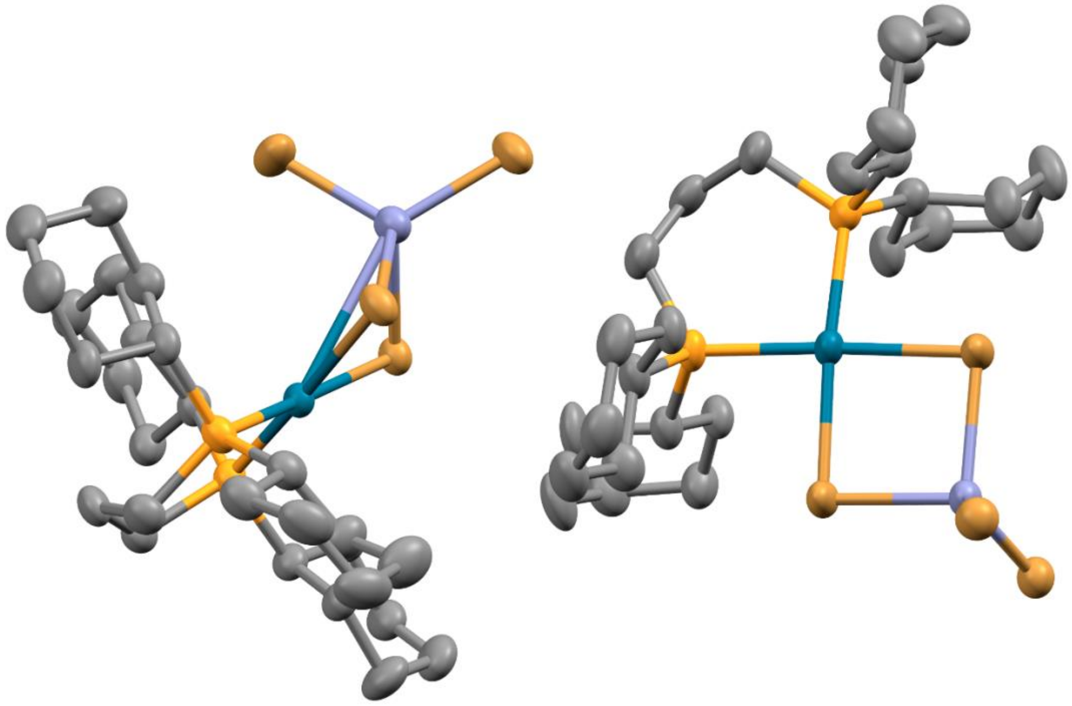

Figure S17. Crystal structure of **3** (both independent molecules in the asymmetric unit). All hydrogen atoms, included solvent molecules, and atoms of the major occupancy orientations are omitted for clarity. Structures represented with Br ions in halide sites.

Computational Methods

DFT calculations for geometry optimisations were carried out using Gaussian 09 (Revision D.01).[9] Geometry optimisations were performed using the M062X[10] density functional, including dispersion corrections described by Grimme's D3 corrections.[11] A functional screening was performed using the following functionals: B3PW91,[12,13] PBE0,[14] M06L[15] and the ωB97x-D,[16] including dispersion corrections described by Grimme's D3 corrections,[11] with added Becke-Johnson dampening where appropriate.[17-20] Metal atoms (Pd and Zn) were described with Stuttgart SDDAll RECPs and associated basis sets,[21] while a hybrid basis set was used for the other atoms: def2-svp (C, H), def2-tzvp(p) (Cl, P).[22]

Geometry optimisation calculations were performed without symmetry constraints and an ultrafine integrations grid (keyword int=ultrafine). The graphical user interface used to visualise the various properties of the optimised structures was GaussView 6.0.16.[23]

Structures were optimised with the multiplicity set as a doublet. Wavefunction stability calculations were performed with $S^2 = 0.885$ and 0.877 for **2a** and **2b** respectively. Geometry optimisations were attempted with quartet and sextet multiplicities for both clusters, however did not lead to convergence of the wavefunctions.

Natural bond order (NBO) analysis was performed with the above described basis sets, using NBO version 6.0.[24,25] QTAIM calculations were performed using the AIMAll software.[26,27] QTAIM parameters were set as follows: electron density cut off as 1×10^{-5}, weak-CP threshold as 0.025, and non-CP threshold as 0.

Functional testing

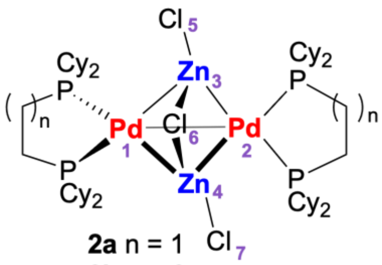

2a n = 1

2b n = 2

Table S2. Functional testing for comparison of bond lengths with single crystal X-ray data for compound **2a**.

Bond	Distance / Å (XRD)	Distance / Å (PBE0)	Distance / Å (B3PW91)	Distance / Å (wB97xD)	Distance / Å (M06L)	Distance / Å (M062X)	Distance / Å (BP86)
Pd1-Pd2	2.7981(4)	2.68078	2.64401	2.68941	2.71983	**2.80356**	2.63688
Zn3-Zn4	2.6818(8)	**2.67448**	2.68832	2.67303	2.65062	2.74238	2.71698
Pd1-Zn3	2.7963(7)	2.55940	2.54764	2.56049	2.63765	**2.92392**	2.55912
Pd2-Zn3	2.4668(6)	2.56169	2.54433	2.59537	2.54799	**2.49926**	2.54075
Pd1-Zn4	2.4589(6)	2.45465	2.44431	**2.46129**	2.49951	2.55448	2.46068
Pd2-Zn4	2.7424(6)	2.85935	2.83665	3.05705	2.73147	**2.73294**	2.76418
Zn4-Cl5	2.1941(12)	**2.20690**	2.20703	2.20693	2.21508	2.23777	2.22072
Zn4-Cl6	2.4571(13)	2.40352	2.41052	2.37101	2.48779	2.52453	**2.44731**
Zn3-Cl6	2.4304(13)	2.48417	2.48802	2.54932	**2.45577**	2.40814	2.49391
Zn3-Cl7	2.2173(13)	2.20803	2.20846	2.20323	**2.21978**	2.23599	2.22196

Table S3. Functional testing for comparison of bond lengths with single crystal X-ray data for compound **2b**.

Bond	Distance / Å (XRD)	Distance / Å (PBE0)	Distance / Å (B3PW91)	Distance / Å (wB97xD)	Distance / Å (M06L)	Distance / Å (M062X)	Distance / Å (BP86)
Pd1-Pd2	2.9033(6)	2.83691	2.78906	2.84816	2.83649	**2.91876**	2.77739
Zn3-Zn4	2.6357(11)	2.65137	2.67119	2.66493	**2.64263**	2.74000	2.70061
Pd1-Zn4	2.4628(9)	**2.48071**	2.47197	2.48669	2.52504	2.52478	2.49414
Pd2-Zn4	2.7080(9)	2.72163	2.69604	2.84214	**2.69991**	2.81763	2.64172
Pd1-Zn3	2.7754(9)	2.59284	2.58953	2.58078	2.67808	**2.81229**	2.61415
Pd2-Zn3	2.4673(8)	2.52809	2.50800	2.55170	2.52344	2.52836	**2.50425**
Zn4-Cl5	2.2041(15)	2.21392	**2.21332**	2.21724	2.22526	2.24359	2.22805
Zn4-Cl6	2.4492(17)	2.38896	2.39277	2.34297	2.42629	2.43133	**2.43763**
Zn3-Cl6	2.4468(17)	2.53782	2.54333	2.68949	2.53343	**2.48673**	2.52935
Zn3-Cl7	2.1914(16)	2.20013	2.20018	**2.19093**	2.21057	2.23170	2.21604

M062X was chosen as the best functional when comparing calculated and experimental bond lengths of both **2a** and **2b**. Hence, this functional was used in further calculations.

Frontier molecular orbitals for 2a and 2b

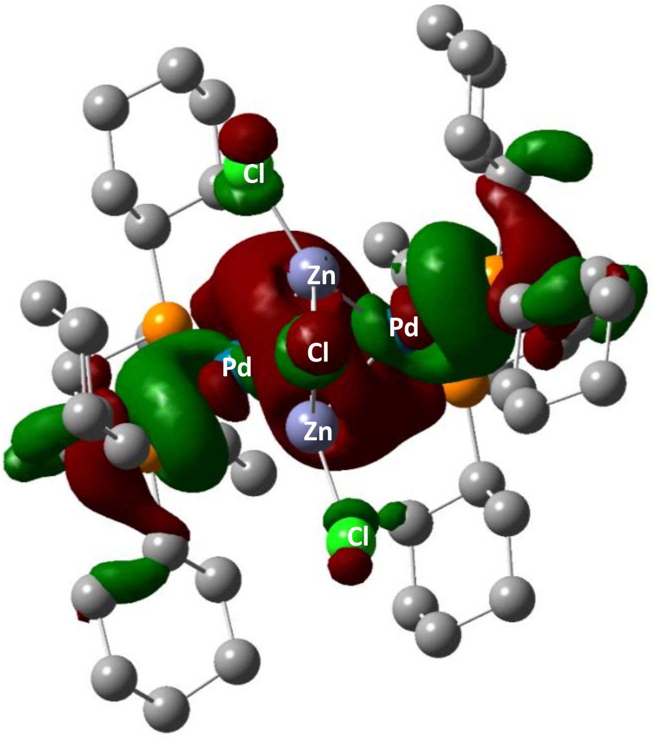

Figure S18. Orbital isosurface (isovalue = 0.02) for the singly occupied molecular orbital (SOMO) on geometry optimised structure of **2a**. Hydrogen atoms omitted for clarity.

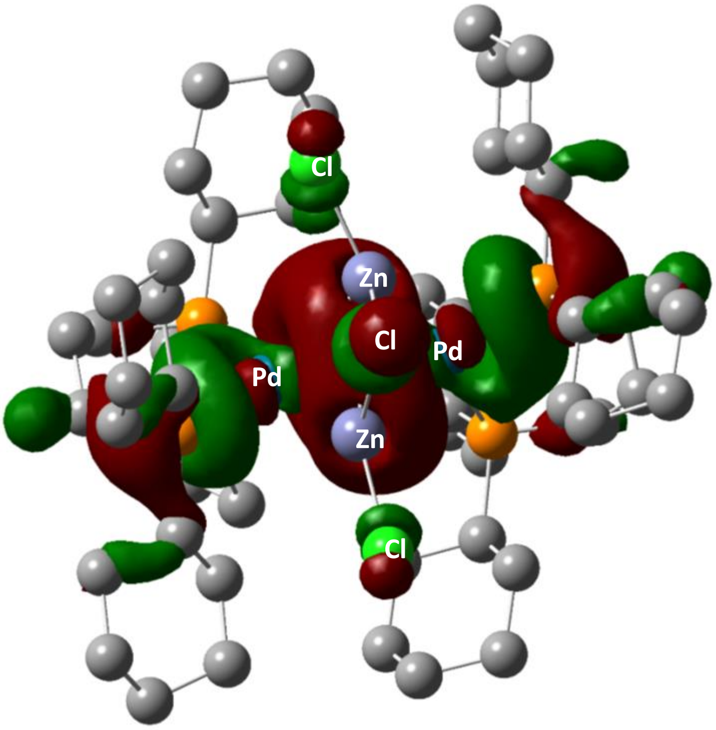

Figure S19. Orbital isosurface (isovalue = 0.02) for the singly occupied molecular orbital (SOMO) on geometry optimised structure of **2b**. Hydrogen atoms omitted for clarity.

Spin density plots for 2a and 2b

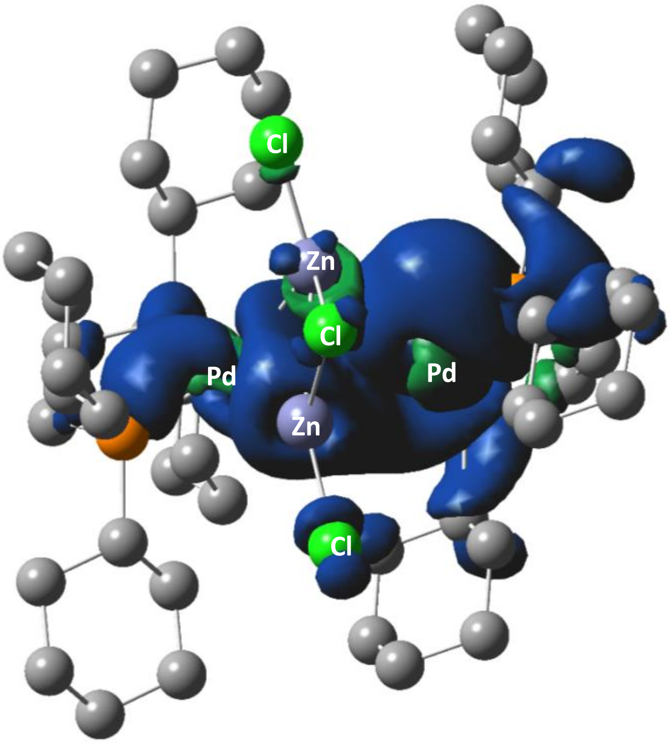

Figure S20. Spin density plot for **2a**. Hydrogen atoms omitted for clarity.

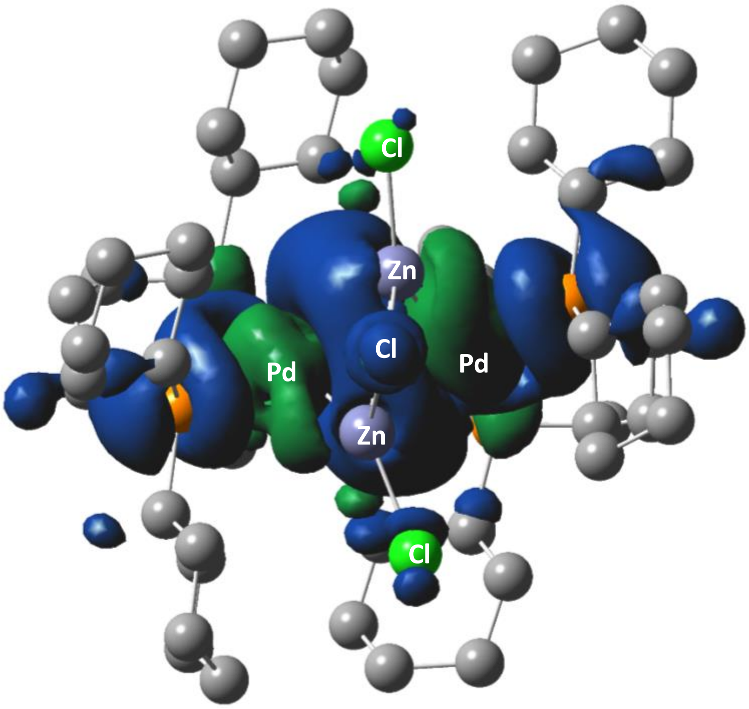

Figure S21. Spin density plot for **2b**. Hydrogen atoms omitted for clarity.

NBO analysis

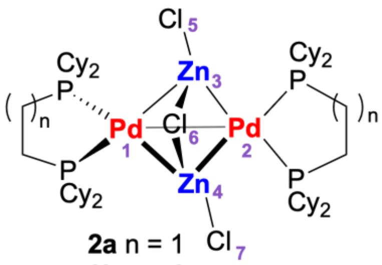

2a n = 1
2b n = 2

Table S4. NPA charges for complex **2a**.

Atom	NPA charge
Pd1	-0.28
Pd2	-0.26
Zn3	0.60
Zn4	0.58
Cl5	-0.40
Cl6	-0.41
Cl7	-0.41

Table S5. Wiberg Bond Indices for complex **2a**.

Bond	WBI
Pd1-Pd2	0.24
Zn3-Zn4	0.073
Pd1-Zn3	0.085
Pd2-Zn3	0.27
Pd1-Zn4	0.29
Pd2-Zn4	0.13
Zn4-Cl5	0.22
Zn4-Cl6	0.10
Zn3-Cl6	0.13
Zn3-Cl7	0.23

Table S6. NPA charges for complex **2b**.

Atom	NPA charge
Pd1	-0.25
Pd2	-0.27
Zn3	0.58
Zn4	0.59
Cl5	-0.40
Cl6	-0.41
Cl7	-0.40

Table S7. Wiberg Bond Indices of complex **2b**.

Bond	WBI
Pd1-Pd2	0.20
Zn3-Zn4	0.076
Pd1-Zn4	0.26
Pd2-Zn4	0.10
Pd1-Zn3	0.11
Pd2-Zn3	0.28
Zn3-Cl5	0.23
Zn3-Cl6	0.12
Zn4-Cl6	0.11
Zn4-Cl7	0.23

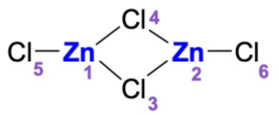

Table S8. NPA charges for Zn_2Cl_4.

Atom	NPA charge
Zn1	1.47
Zn2	1.47
Cl3	-0.73
Cl4	-0.74
Cl5	-0.74
Cl6	-0.73

Table S9. Wiberg Bond Indices for Zn_2Cl_4.

Bond	WBI
Zn1-Cl3	0.21
Zn1-Cl4	0.21
Zn1-Cl5	0.44
Zn2-Cl3	0.21
Zn2-Cl4	0.21
Zn2-Cl6	0.44

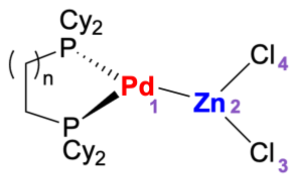

Table S10. NPA charges for fragment $L_2PdZnCl_2$ (L_2 = dcpe).

Atom	NPA charge
Pd1	-0.035
Zn2	1.33
Cl3	-0.77
Cl4	-0.72

Table S11. Wiberg Bond Indices for fragment $L_2PdZnCl_2$ (L_2 = dcpe).

Bond	WBI
Pd1-Zn2	0.21
Zn2-Cl3	0.33
Zn2-Cl4	0.22
Pd1-Cl4	0.18

Table S12. NPA charges for fragment $L_2PdZnCl_2$ (L_2 = dcpp).

Atom	NPA charge
Pd1	-0.056
Zn2	1.34
Cl3	-0.77
Cl4	-0.73

Table S13. Wiberg Bond Indices for fragment $L_2PdZnCl_2$ (L_2 = dcpp).

Bond	WBI
Pd1-Zn2	0.22
Zn2-Cl3	0.33
Zn2-Cl4	0.22
Pd1-Cl4	0.17

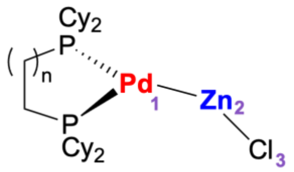

Table S14. NPA charges for fragment $L_2PdZnCl$ (L_2 = dcpe).

Atom	NPA charge
Pd1	-0.18
Zn2	0.79
Cl3	-0.83

Table S15. Wiberg Bond Indices for fragment $L_2PdZnCl$ (L_2 = dcpe).

Bond	WBI
Pd1-Zn2	0.55
Zn2-Cl3	0.20

Table S16. NPA charges for fragment $L_2PdZnCl$ (L_2 = dcpp).

Atom	NPA charge
Pd1	-0.19
Zn2	0.80
Cl3	-0.82

Table S17. Wiberg Bond Indices for fragment $L_2PdZnCl$ (L_2 = dcpp).

Bond	WBI
Pd1-Zn2	0.54
Zn2-Cl3	0.21

QTAIM analysis

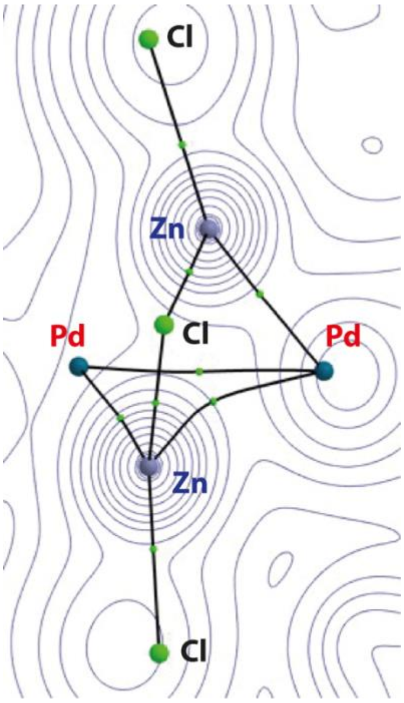

Bond	ρ	Δ²ρ
Pd1-Pd2	0.037	+0.075
Zn3-Zn4	n/a	n/a
Pd1-Zn3	n/a	n/a
Pd2-Zn3	0.056	+0.041
Pd1-Zn4	0.052	+0.051
Pd2-Zn4	0.035	+0.044
Zn4-Cl5	0.072	+0.27
Zn4-Cl6	0.040	+0.12
Zn3-Cl6	0.050	+0.17
Zn3-Cl7	0.072	+0.27

Figure S22. Left: QTAIM contour plot of ρ for **2a**. Bond critical points (ρ) are shown as green dots. Palladium, zinc, and chlorine atoms are in blue, purple, and green respectively. Right: QTAIM data for bond critical points for complex **2a**. All values are in units of e bohr^{-3} (ρ) and e bohr^{-5} ($\Delta^2\rho$).

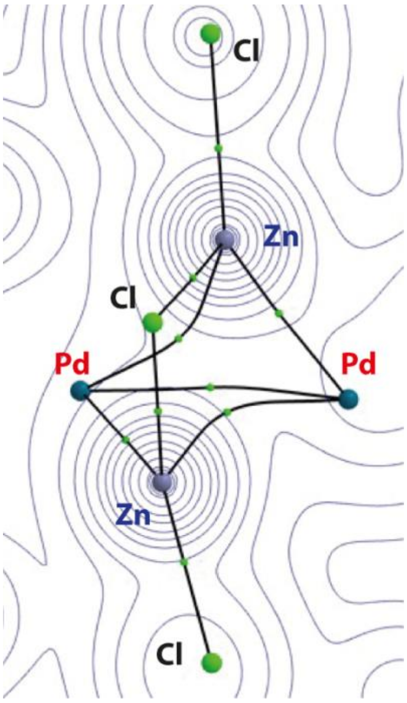

Bond	ρ	∇²ρ
Pd1-Pd2	0.032	+0.058
Zn3-Zn4	n/a	n/a
Pd1-Zn3	0.031	+0.037
Pd2-Zn3	0.054	+0.062
Pd1-Zn4	0.053	+0.066
Pd2-Zn4	0.031	+0.034
Zn4-Cl5	0.071	+0.26
Zn4-Cl6	0.048	+0.16
Zn3-Cl6	0.043	+0.13
Zn3-Cl7	0.073	+0.27

Figure S23. Left: QTAIM contour plots of ρ for **2b**. Bond critical points (ρ) are shown as green dots. Palladium, zinc, and chlorine atoms are in blue, purple, and green respectively. Right: QTAIM data for bond critical points for complex **2b**. All values are in units of e bohr^{-3} (ρ) and e bohr^{-5} ($\nabla^2\rho$).

References

(1) Garçon, M.; Mun, N. W.; White, A. J. P.; Crimmin, M. R. Palladium-Catalysed C–H Bond Zincation of Arenes: Scope, Mechanism, and the Role of Heterometallic Intermediates. *Angew. Chem. Int. Ed.* **2021**, *60* (11), 6145-6153.

(2) Anderson, G. K.; Lin, M.; Sen, A.; Gretz, E. Bis(Benzonitrile)Dichloro Complexes of Palladium and Platinum. In *Inorganic Syntheses*; Inorganic Syntheses; 1990, pp 60-63.

(3) Wang, J.-x.; Jia, X.; Meng, T.; Xin, L. Rapid and Solvent-Free Synthesis of Homoallyl or Homopropargyl Alcohols Mediated by Zinc Powder. *Synth.* **2005**, *2005* (17), 2838-2844.

(4) Dolomanov, O. V.; Bourhis, L. J.; Gildea, R. J.; Howard, J. A. K.; Puschmann, H. OLEX2: a complete structure solution, refinement and analysis program. *J. Appl. Cryst.* **2009**, *42*, 339-341.

(5) Sheldrick, G. M. SHELXT – Integrated space-group and crystal-structure determination. *Acta Crystallogr. Sect. A Found. Adv.* **2015**, *71*, 3-8.

(6) Sheldrick, G. M. Crystal structure refinement with SHELXL *Acta Crystallogr. Sect. C Struct. Chem.* **2015**, *71*, 3-8.

(7) Krzystek, J.; Sienkiewicz, A.; Pardi, L.; Brunel, L. C. DPPH as a Standard for High-Field EPR. *J. Magn. Reson.* **1997**, *125* (1), 207-211.

(8) Stoll, S.; Schweiger, A. EasySpin, a comprehensive software package for spectral simulation and analysis in EPR. *J. Magn. Reson.* **2006**, *178* (1), 42-55.

(9) Frisch, M. J.; Trucks, G. W.; Schlegel, H. B.; Scuseria, G. E.; Robb, M. A.; Cheeseman, J. R.; Scalmani, G.; Barone, V.; Mennucci, B.; Petersson, G. A.; Nakatsuji, H.; Caricato, M.; Li, X.; Hratchian, H. P.; Izmaylov, A. F.; Bloino, J.; Zheng, G.; Sonnenberg, J. L.; Hada, M.; Ehara, M.; Toyota, K.; Fukuda, R.; Hasegawa, J.; Ishida, M.; Nakajima, T.; Honda, Y.; Kitao, O.; Nakai, H.; Vreven, T.; Montgomery, J. A., Jr.; Peralta, J. E.; Ogliaro, F.; Bearpark, M.; Heyd, J. J.; Brothers, E.; Kudin, K. N.; Staroverov, V. N.; Kobayashi, R.; Normand, J.; Raghavachari, K.; Rendell, A.; Burant, J. C.; Iyengar, S. S.; Tomasi, J.; Cossi, M.; Rega, N.; Millam, J. M.; Klene, M.; Knox, J. E.; Cross, J. B.; Bakken, V.; Adamo, C.; Jaramillo, J.; Gomperts, R.; Stratmann, R. E.; Yazyev, O.; Austin, A. J.; Cammi, R.; Pomelli, C.; Ochterski, J. W.; Martin, R. L.; Morokuma, K.; Zakrzewski, V. G.; Voth, G. A.; Salvador, P.; Dannenberg, J. J.; Dapprich, S.; Daniels, A. D.; Farkas, Ö.; Foresman, J. B.; Ortiz, J. V.; Cioslowski, J.; Fox, D. J. Gaussian 09, Revision D.01; Gaussian Inc., Wallingford, CT, **2009**.

(10) Zhao, Y.; Truhlar, D. G. The M06 suite of density functionals for main group thermochemistry, thermochemical kinetics, noncovalent interactions, excited states, and transition elements: two new functionals and systematic testing of four M06-class functionals and 12 other functionals. *Theor. Chem. Acc.* **2008**, *120* (1), 215-241.

(11) Grimme, S.; Antony, J.; Ehrlich, S.; Krieg, H. A consistent and accurate ab initio parametrization of density functional dispersion correction (DFT-D) for the 94 elements H-Pu. *J. Chem. Phys.* **2010**, *132* (15), 154104.

(12) Perdew, J. P.; Chevary, J. A.; Vosko, S. H.; Jackson, K. A.; Pederson, M. R.; Singh, D. J.; Fiolhais, C. Atoms, molecules, solids, and surfaces: Applications of the generalized gradient approximation for exchange and correlation. *Phys. Rev. B* **1992**, *46* (11), 6671-6687.

(13) Perdew, J. P.; Chevary, J. A.; Vosko, S. H.; Jackson, K. A.; Pederson, M. R.; Singh, D. J.; Fiolhais, C. Erratum: Atoms, molecules, solids, and surfaces: Applications of the generalized gradient approximation for exchange and correlation. *Phys. Rev. B* **1993**, *48* (7), 4978-4978.

(14) Adamo, C.; Cossi, M.; Barone, V. An accurate density functional method for the study of magnetic properties: the PBE0 model. *J. Mol. Struct. Theochem* **1999**, *493* (1), 145-157.

(15) Zhao, Y.; Truhlar, D. G. A new local density functional for main-group thermochemistry, transition metal bonding, thermochemical kinetics, and noncovalent interactions. *J. Chem. Phys.* **2006**, *125* (19), 194101.

(16) Chai, J.-D.; Head-Gordon, M. Long-range corrected hybrid density functionals with damped atom–atom dispersion corrections. *Phys. Chem. Chem. Phys.* **2008**, *10* (44), 6615-6620.

(17) Becke, A. D.; Johnson, E. R. A density-functional model of the dispersion interaction. *J. Chem. Phys.* **2005**, *123* (15), 154101.

(18) Johnson, E. R.; Becke, A. D. A post-Hartree–Fock model of intermolecular interactions. *J. Chem. Phys.* **2005**, *123* (2), 024101.

(19) Johnson, E. R.; Becke, A. D. A post-Hartree-Fock model of intermolecular interactions: Inclusion of higher-order corrections. *J. Chem. Phys.* **2006**, *124* (17), 174104.

(20) Grimme, S.; Ehrlich, S.; Goerigk, L. Effect of the damping function in dispersion corrected density functional theory. *J. Comput. Chem.* **2011**, *32* (7), 1456-1465.

(21) Andrae, D.; Häußermann, U.; Dolg, M.; Stoll, H.; Preuß, H. Energy-adjustedab initio pseudopotentials for the second and third row transition elements. *Theor. Chim. Acta* **1990**, *77* (2), 123-141.

(22) Weigend, F.; Ahlrichs, R. Balanced basis sets of split valence, triple zeta valence and quadruple zeta valence quality for H to Rn: Design and assessment of accuracy. *Phys. Chem. Chem. Phys.* **2005**, *7* (18), 3297-3305.

(23) Dennington, R.; Keith, T.; Milliam, J. GaussView 6.0; Semichem Inc., Shawnee Mission, KS, **2019**.

(24) NBO 6.0. Glendening, E. D.; Badenhoop, J. K.; Reed, A. E.; Carpenter, J. E.; Bohmann, J. A.; Morales, C. M.; Landis, C. R.; Weinhold, F. Theoretical Chemistry Institute, University of Wisconsin, Madison (2013).

(25) Glendening, E. D.; Landis, C. R.; Weinhold, F. NBO 6.0: Natural bond orbital analysis program. *J. Comput. Chem.* **2013**, *34* (16), 1429-1437.

(26) AIMALL (Version 19.10.12). Keith, T. A. TK Gristmill Software, Overland Park KS, USA (2019).

(27) Cortés-Guzmán, F.; Bader, R. F. W. Complementarity of QTAIM and MO theory in the study of bonding in donor–acceptor complexes. *Coord. Chem. Rev.* **2005**, *249* (5), 633-662.
